# Single Nucleotide Polymorphisms within Interferon Signaling Pathway Genes Are Associated with Colorectal Cancer Susceptibility and Survival

**DOI:** 10.1371/journal.pone.0111061

**Published:** 2014-10-28

**Authors:** Shun Lu, Barbara Pardini, Bowang Cheng, Alessio Naccarati, Stefanie Huhn, Veronika Vymetalkova, Ludmila Vodickova, Thomas Buchler, Kari Hemminki, Pavel Vodicka, Asta Försti

**Affiliations:** 1 Division of Molecular Genetic Epidemiology, German Cancer Research Center (DKFZ), Heidelberg, Germany; 2 Human Genetics Foundation (HuGeF), Turin, Italy; 3 Department of Molecular Biology of Cancer, Institute of Experimental Medicine, Academy of Sciences of the Czech Republic, Prague, Czech Republic; 4 Institute of Biology and Medical Genetics, 1st Medical Faculty, Charles University, Prague, Czech Republic; 5 Biomedical Centre, Faculty of Medicine in Pilsen, Charles University in Prague, Pilsen, Czech Republic; 6 Department of Oncology, Thomayer Hospital, Prague, Czech Republic; 7 Center of Primary Health Care Research, Clinical Research Center, Lund University, Malmö, Sweden; Institut Curie, France

## Abstract

Interferon (IFN) signaling has been suggested to play an important role in colorectal carcinogenesis. Our study aimed to examine potentially functional genetic variants in interferon regulatory factor 3 (*IRF3*), *IRF5*, *IRF7*, type I and type II *IFN* and their receptor genes with respect to colorectal cancer (CRC) risk and clinical outcome. Altogether 74 single nucleotide polymorphisms (SNPs) were covered by the 34 SNPs genotyped in a hospital-based case-control study of 1327 CRC cases and 758 healthy controls from the Czech Republic. We also analyzed these SNPs in relation to overall survival and event-free survival in a subgroup of 483 patients. Seven SNPs in *IFNA1*, *IFNA13*, *IFNA21*, *IFNK*, *IFNAR1* and *IFNGR1* were associated with CRC risk. After multiple testing correction, the associations with the SNPs rs2856968 (*IFNAR1*) and rs2234711 (*IFNGR1*) remained formally significant (*P* = 0.0015 and *P*<0.0001, respectively). Multivariable survival analyses showed that the SNP rs6475526 (*IFNA7/IFNA14*) was associated with overall survival of the patients (*P* = 0.041 and event-free survival among patients without distant metastasis at the time of diagnosis, *P* = 0.034). The hazard ratios (HRs) for rs6475526 remained statistically significant even after adjustment for age, gender, grade and stage (*P* = 0.029 and *P* = 0.036, respectively), suggesting that rs6475526 is an independent prognostic marker for CRC. Our data suggest that genetic variation in the IFN signaling pathway genes may play a role in the etiology and survival of CRC and further studies are warranted.

## Introduction

Colorectal cancer (CRC) is an important contributor to cancer incidence and death, with more than 1.3 million new cases resulting in about 694,000 deaths in 2012 worldwide (http://globocan.iarc.fr/Default.aspx). Nutrition, lifestyle factors and environment [1], as well as genetic events have been implicated both in the causation of CRC and survival of patients after diagnosis of CRC [2], [3]. So far, 50 single nucleotide polymorphisms (SNPs) located in 40 loci have been associated with the risk of CRC by genome-wide association studies (GWASs, http://www.genome.gov/gwastudies/, [4]–[6]). Although molecular markers have been suggested for follow-up of treated CRC patients, their complete clinical application is under evaluation. Clinicopathologic stage is still the main prognostic marker used in the clinical practice.

Inflammatory responses play a crucial role in colorectal carcinogenesis. Several studies, although not any GWAS, have reported SNPs in immune-related genes to be associated with CRC risk or prognosis [7]–[10]. Interferons (IFNs) are immune-related proteins produced and released by host cells in response to the presence of pathogens. IFN-mediated signaling has a diverse range of functions, including antiviral and antimicrobial response, antiproliferation, immunomodulation and apoptosis [11], [12]. There are two main classes of IFNs, type I and type II. The two type I IFNs, IFNA and IFNB, have been reported to have an effect on tumor suppression and antiviral immune defense through induction of p53 responses [13]. IFNG, the only type II IFN, has been suggested to play a vital role in the disruption of the intestinal epithelial barrier function [14], [15]. It has also been identified as an important modulator of immune-related genes, such as toll-like receptor 3 (*TLR3*), the gene which showed association with CRC survival in our previous study [8]. Interferon regulatory factors (IRFs) regulate IFNs and some IFN-inducible oncogenes by serving as transcription mediators of pathogens and IFN-induced signaling pathways. Interferon receptors are essential for IFNs to exert their biological effects [11], [12]. All type I IFNs bind to a receptor composed of two subunits, IFNAR1 and IFNAR2, while the type II interferon IFNG binds to another dimeric receptor composed of IFNGR1 and IFNGR2.

So far, few studies have investigated the association between genetic variants in the IFN signaling pathway and CRC. A previous study examined genetic variation in *IFNG, IFNGR1, IFNGR2* and IRF1-9 with the risk and survival of colon and rectal cancer [16]. In that study, tagSNP approach was applied; several SNPs in *IRFs, IFNG* and its receptors were found to be associated with CRC risk or survival [16]. To further explore the role of genetic variants in the IFN signaling pathway genes in CRC, we genotyped a set of potentially functional SNPs in the *IRF3, IRF5, IRF7, IFNA, IFNB, IFNE, IFNK, IFNW, IFNG, IFNAR1, IFNAR2, IFNGR1* and *IFNGR2* genes in a case-control study of 1327 CRC patients and 758 healthy controls from the Czech Republic and evaluated their association with CRC susceptibility, progression, and prognosis.

## Materials and Methods

### Ethics statement

All participants gave a written informed consent to the use of their samples for research purpose. The study was approved by the ethical committees of the participating institutes, the Institute of Experimental Medicine, Academy of Sciences of the Czech Republic, Prague, Czech Republic and the Institute for Clinical and Experimental Medicine and Faculty Thomayer Hospital, Prague, Czech Republic.

### Study population

The case group contained 1327 CRC patients recruited between the years 2004 and 2010 by several oncological departments in the Czech Republic [17]. Their mean age (± standard deviation) was 62.1 (±10.7) years, and 61.7% of them were men. The patients showed positive colonoscopic results for malignancy, histologically confirmed as colon or rectal carcinomas. Patients who met the Amsterdam criteria I or II for hereditary nonpolyposis colorectal cancer were not included in the study [18]. General information about gender and age at diagnosis was available for all patients. For 483 consecutively recruited, incident cases diagnosed between 2003 and 2010, clinical data at the time of diagnosis, including location of the tumor (colon/rectum), International Union against Cancer (UICC) TNM stage classification [size or direct extent of the primary tumor (T), degree of spread to regional lymph nodes (N), presence of metastasis (M)] and grade were available (Table 1). Information about distant metastasis, relapse and date of death was also collected, with a follow-up until August 31, 2011.

**Table 1:** Characteristics of the 483 newly diagnosed Czech colorectal cancer patients.

Characteristics	No. (%)
Age at diagnosis, mean (range, SD)	63.5 (27–89, 10.34)
<65	243 (50.31)
≥65	240 (49.69)
Gender	
Female	180 (37.27)
Male	303 (62.73)
Diagnosis	
Colon	298 (61.70)
Rectum	185 (38.30)
Grade	
1, 2	309 (63.98)
3, 4	105 (21.74)
Missing	69 (14.29)
T	
T1, T2	88 (18.22)
T3, T4	351 (72.67)
Missing	44 (9.11)
N	
N0	216 (44.72)
N1, N2	191 (39.54)
Missing	76 (15.73)
M	
M0	325 (67.29)
M1	126 (26.09)
Missing	32 (6.22)
TNM stage	
Stage I	55 (11.39)
Stage II	128 (26.50)
Stage III	123 (25.47)
Stage IV	126 (26.09)
Missing	51 (10.56)
Relapse	
Yes	91 (18.84)
No	392 (81.16)
Death	
Yes	245 (50.72)
No	238 (49.28)

No., number of patients; T, size or direct extent of the primary tumor; N, degree of spread to regional lymph nodes; M, presence of metastasis.

The control group contained 758 healthy individuals recruited by a blood-donor center in one hospital in Prague [19]. These disease-free individuals represent the general population of the Czech Republic, which has a genetically quite uniform population [20]–[22]. Their mean age (± standard deviation) was 45.6 (±8.3) years, and 56.2% of them were men.

### SNP selection

20 candidate genes were selected from IFN signaling pathway based on their suggested functional role in CRC causation and survival, including *IFNA (1, 2, 4, 5, 7, 8, 13, 16, 17, and 21), IFNB1, IFNK, IFNW1, IRF3, IRF5, IRF7, IFNAR1, IFNAR2, IFNGR1*, and *IFNGR2* [8], [13]–[15], [23]–[28]. A total of 34 SNPs, which captured 74 potentially functional SNPs, were selected for genotyping in these genes from the International HapMap Project (http://hapmap.ncbi.nlm.nih.gov) and the NCBI database (http://www.ncbi.nlm.nih.gov) (Table 2) based on the following criteria: minor allele frequency (MAF) ≥10% in Europeans; location within the coding region (non-synonymous SNPs), the 5′ and 3′ untranslated regions (UTRs) and the promoter (up to approximately 1 kb from the transcription start site); pairwise linkage disequilibrium (LD, r^2^≤0.80) between the SNPs in Utah residents with Northern and Western European ancestry from the CEPH collection (CEU). SNPnexus (http://snp-nexus.org/) was used to predict functional consequences of the selected SNPs. For the SNPs rs2856968, rs2243711 and rs6475526 (including SNPs captured by these SNPs), which associated with CRC risk or survival, we also used additional web-based tools [HaploReg v2 (http://www.broadinstitute.org) and SNPinfo Web Server (http://snpinfo.niehs.nih.gov/cgi-bin/snpinfo/snpfunc.cgi)] to predict their effects on potential regulatory elements.

**Table 2:** Polymorphisms evaluated in this study.

*Gene*	Genotyped SNP ID	Alleles (major/minor)	Chromosome	Position	Location	MAF1 (NCBI)	MAF1 in controls	SNP captured with r^2^≥0.80^2^			
								Gene	SNP ID	Position	Location
*IFNB1*	rs1424855	C/G	9p21.3	21078815	5' near gene	0.375	0.353				
	rs10964859	C/G	9p21.3	21140672	3' UTR	0.394	0.343				
*IFNW1*	rs10757189	G/A	9p21.3	21142604	5' near gene	0.311	0.264	IFNW1	rs10511694	21143021	5' near gene
*IFNA21*	rs2939	T/C	9p21.3	21166004	3' UTR	0.125	0.204	IFNA4 IFNA17 IFNA4 IFNA4 IFNA7 IFNA10 IFNA16 IFNA16 IFNA17 IFNA17 IFNA17 IFNA14 IFNA14 IFNA14 IFNA14 IFNA13 IFNA4 IFNA4 IFNA4 IFNA7 IFNA17	rs3750479 rs7858057 rs7035639 rs10964896 rs10757199 rs12555631 rs1834247 rs1424854 rs10964918 rs10964920 rs1831391 rs4628333 rs12553763 rs12551190 rs10964932 rs637949 rs12236048 rs10964898 rs10964899 rs10811502 rs7868588	21186932 21228760 21189263 21188208 21202357 21206428 21217536 21217850 21228286 21229185 21229328 21238963 21241450 21241490 21241857 21369316 21186255 21188271 21188353 21203009 21227087	3' UTR Intron variant 5' near gene 5' near gene 5' near gene 3' UTR Intron variant Intron variant Intron variant Intron variant Intron variant Intron variant 5' near gene 5' near gene 5' near gene 5' near gene 3' near gene 5' near gene 5' near gene 5' near gene 3' near gene
*IFNA21*	rs12376071	A/G	9p21.3	21166902	5' near gene	0.317	0.305				
*IFNA21*	rs7047687	A/C	9p21.3	21167652	5' near gene	0.425	0.487	IFNA21 IFNA4	rs2891157 rs7870840	21168307 21187929	5' near gene 5' near gene
*IFNA4*	rs2383183	T/C	9p21.3	21187700	5' near gene	0.1	0.122				
*IFNA16*	rs10964912	A/C	9p21.3	21218096	5' near gene	0.259	0.222				
*IFNA17*	rs7873404	T/C	9p21.3	21228497	5' near gene	0.183	0.233				
*IFNA7/IFN14*	rs6475526^3^	C/T	9p21.3	21242162	5' near gene	0.376	0.352	IFNA7 IFNA7	rs7046208 rs7045980	21202409 21202411	5 near gene 5' near gene
*IFNA5*	rs12156640	G/A	9p21.3	21306241	5' near gene	0.1	0.11				
*IFNA13*	rs641734	A/T	9p21.3	21368927	5' near gene	0.175	0.194	IFNA6 IFNA13 IFNA5 IFNA6 IFNA6	rs2990144 rs653778 rs7031048 rs2988573 rs614541	21350079 21368098 21306319 21350621 21352863	3' UTR 5' UTR 5' near gene Synonymous variant 5' near gene
*IFNA2*	rs10120977	A/G	9p21.3	21384363	3' UTR	0.248	0.209				
	rs12553575	A/G	9p21.3	21408498	5' near gene	0.158	0.144				
*IFNA8*	rs10738592	C/T	9p21.3	21408516	5' near gene	0.475	0.486				
	rs10811536	T/C	9p21.3	21408693	5' near gene	0.233	0.2	IFNA8	rs10811537	21408825	5' near gene
*IFNA1*	rs33965070	C/G	9p21.3	21440994	missense	0.182	0.068				
*IFNK*	rs700782	G/A	9p21.3	27526047	3' UTR	0.243	0.21				
*IRF3*	rs2304204	A/G	19q13	50169020	5' UTR	0.221	0.303	IRF3	rs2304205	49665670	5' near gene
	rs2070197	T/C	7q23	128589000	3' UTR	0.15	0.107				
*IRF5*	rs11770589	G/A	7q23	128589488	3' UTR	0.375	0.476				
*IRF5*	rs1874327^3^	T/A	7q23	128945322	intron	0.400	0.358	IRF5 IRF5 IRF5	rs10954214rs10954213 rs3757385	128949579128949373 128937250	3'UTR 3' UTR 5' near gene
*IRF7*	rs1061502	A/G	11p15.5	614318	missense	0.283	0.231	IRF7 IRF7 IRF7 IRF7 IRF7	rs7943546 rs1061505 rs1055382 rs12805435 rs1131665	612148 613297 612382 612355 613208	3' UTR synonymous codon 3' near gene 3' near gene missense
*IFNAR2*	rs1131668	G/A	21	33262573	missense	0.332	0.326	IFNAR2	rs1051393	33241950	Missense
*IFNAR1*	rs2856968^3^	A/G	21	33325676	intron	0.424	0.345	IFNAR1 IFNAR1 IFNAR1	rs17875752 rs17875753 rs2843710	33324192 33324196 33324402	5' near gene 5' near gene 5' near gene
*IFNAR1*	rs2850015	C/T	21	34697264	5' UTR	0.308	0.296				
*IFNAR1*	rs2257167	G/C	21	34715699	missense	0.127	0.14				
*IFNAR1*	rs2834202	A/G	21	34730954	3' UTR	0.261	0.218				
*IFNGR1*	rs2234711	T/C	6	137540520	5' UTR	0.353	0.375				
*IFNGR1*	rs17181457	C/T	6	137540536	5' UTR	0.117	0.078				
*IFNGR1*	rs1327474	G/A	6	137541075	5' near gene	0.398	0.447				
*IFNGR2*	rs17882748	T/C	21	34775721	5' UTR	0.413	0.49				
*IFNGR2*	rs9808753	A/G	21	34787312	missense	0.142	0.12				
*IFNGR2*	rs1059293	T/C	21	34809693	3' UTR	0.478	0.445				

1 Minor allele frequency (MAF) based on Utah residents with Northern and Western European ancestry from the CEPH collection in the HapMap project.

2 Pairwise linkage disequilibrium (r^2^) was calculated for the SNPs with MAF≥10% within the regions of interest based on Utah residents with Northern and Western European ancestry from the CEPH collection in the HapMap project.

3 Because no assays were available for the potentially functionally SNPs, the SNPs rs6475526, rs1874327 and rs2856968, respectively, were genotyped instead.

### Genotyping

In this project, whole genome amplified (WGA) DNA from peripheral blood leukocytes was used [29], [30]. The genotyping was performed blinded by the case–control status of each sample. The KASP allelic discrimination method (LGCgenomics, Middelsex, UK) was used to genotype the selected SNPs. DNA amplification was performed according to the LGCgenomics' PCR conditions. Genotype detection was performed using an ABI PRISM 7900HT Sequence Detection system with SDS2.4 software (Applied Biosystems). The sample set contained 138 duplicated samples as quality controls. The genotype correlation between the duplicate samples was>99%. Genotype call rate ranged between 97.0 and 99.5%.

### Statistical analysis

The observed genotype frequencies in the controls were tested for Hardy–Weinberg equilibrium (HWE) using the chi-square test. Odds ratios (ORs) and 95% confidence intervals (CIs) for associations between genotypes and CRC risk were calculated by logistic regression (PROC LOGISTIC, SAS Version 9.2; SAS Institute, Cary, NC), and adjusted for age and gender. To account for multiple testing, the SNP Spectral Deposition (SNPSpD) method for multilocus analyses was applied [24], [31]_ENREF_31. For a polymorphism with a variant allele frequency between 10 and 50%, the study had greater than 90% power to detect an OR of 1.50 at a significance level of 0.05 (PS—software for power and sample size calculation, http://biostat.mc.vanderbilt.edu/twiki/bin/view/Main/PowerSampleSize). In this study, we analyzed overall survival in the group of 483 consecutively recruited, incident CRC cases diagnosed between 2003 and 2010, using the date of death or end of the study (August 31, 2011) as the end point of follow-up. Median follow-up time for the 483 patients was 58 months. For event-free survival in patients with non-metastatic disease at the time of diagnosis (n = 325), date of distant metastasis, relapse, death or end of the study was used as the end point of follow-up. Event-free survival was defined as the time from surgery to the occurrence of distant metastasis, recurrence or death, whichever came first. Median follow-up time was 55 months. The survival curves for overall and event-free survival were derived by the Kaplan–Meier method (PROC LIFETEST, SAS Version 9.2) and compared using log-rank test. The relative risk of death was estimated as hazard ratio (HR) using Cox regression (PROC PHREG, SAS Version 9.2). Multivariable survival analyses were adjusted for age, gender, T, N, M, TNM stage and grade separately, and in a final model for age, gender, tumor location, TNM stage and grade. Covariables were stratified for in the analysis if they did not meet the proportional hazards assumption.

## Results

Altogether, 74 SNPs with MAF ≥10% in the CEU population were located within the regions of interest (promoter, 5′ and 3′UTR, non-synonymous SNPs) of the 20 genes *IFNA (1, 2, 4, 5, 7, 8, 13, 16, 17, and 21), IFNB1, IFNK, IFNW1, IRF3, IRF5, IRF7, IFNAR1, IFNAR2, IFNGR1* and *IFNGR2*. From these, 34 SNPs were selected for genotyping based on LD (r^2^≤0.80) (Table 2). All *IFNA* genes as well as the *IFNB1*, *IFNK* and *IFNW1* genes are located at the same locus at 9p21.3. Thus, although the SNPs were selected based on their potential functional effect on a specific gene, they may capture, and thus give information, about additional SNPs and other genes at the same locus, as shown in Table 2 and Figure S1. The genotype distribution of all 34 genotyped polymorphisms was consistent with HWE in the control group (*P*>0.05). The MAFs in the control population were similar to the ones reported by the HapMap project for the CEU population (Table 2).

### Seven SNPs were associated with CRC susceptibility

Minor allele carriers of the *IFNA13* promoter SNP rs641734, and *IFNA21* 3′UTR SNP rs2939, had a decreased risk of CRC, while the minor allele carriers of the *IFNA1* missense SNP rs33965070, *IFNK* 3′UTR SNP rs700782, *IFNAR1* 3′UTR SNP rs2834202, *IFNAR1* SNP rs2856968, which was genotyped instead of the promoter SNPs in the same gene, and *IFNGR1* 5′UTR SNP rs2234711 had an increased risk of CRC (Table 3). These associations did not differ by tumor location at colon or rectum (data not shown). To correct for multiple testing, we used the SNPSpD approach. The study-wise effective number of independent markers M_eff_ was calculated to be 27, which gave the significance threshold of 0.0019. Thus, the associations with the SNPs rs2856968 (*IFNAR1*) and rs2234711 (*IFNGR1*) remained formally significant (*P* = 0.0015 and *P*<0.0001, respectively). The other genotyped SNPs were not associated with CRC risk (Table S1).

**Table 3:** Associations between candidate SNPs and colorectal cancer susceptibility.

Gene	SNP rs#	Genotype	Case No. (%)^1^	Control No. (%)^1^	OR (95% CI)	*P* value^2^
IFNA1	rs33965070	CC	1060 (82.23)	640(86.37)	1	
		CG	229 (17.77)	101 (13.63)	**1.37 (1.06–1.75)**	**0.015**
		GG	0	0		
		CG+GG	229 (17.77)	101 (13.63)	**1.37 (1.06–1.75)**	**0.015**
IFNA13	rs641734	CC	900 (69.39)	473 (64.35)	1	
		CT	358 (27.60)	239 (32.52)	0.79 (0.65–0.96)	**0.018**
		TT	39 (3.01)	23 (3.13)	0.89 (0.53–1.52)	0.668
		TC+CC	397 (30.61)	262 (35.65)	**0.80 (0.66–0.97)**	**0.024**
IFNA21	rs2939	TT	882 (67.90)	466 (62.38)	1	
		CT	383 (29.48)	257 (34.40)	**0.79 (0.65–0.95)**	**0.016**
		CC	34 (2.62)	24 (3.21)	0.75 (0.44–1.28)	0.288
		CT+CC	417 (32.10)	281 (37.62)	**0.79 (0.65–0.95)**	**0.012**
IFNK	rs700782	GG	743 (57.15)	467 (62.18)	1	
		AG	484 (37.23)	252 (33.56)	1.20 (1.00–1.47)	0.054
		AA	73 (5.62)	32 (4.26)	**1.43 (0.93–2.22)**	**0.102**
		AG+AA	557 (42.85)	284 (37.82)	1.23 (1.03–1.49)	0.023
IFNAR1	rs2834202	AA	715 (56.08)	448 (62.22)	1	
		AG	481 (37.73)	230 (31.94)	**1.32 (1.08–1.59)**	**0.007**
		GG	79 (6.20)	42 (5.83)	1.18 (0.79–1.75)	0.412
		AG+GG	560 (43.92)	272 (37.78)	**1.30 (1.08–1.56)**	**0.007**
IFNAR1	rs2856968	AA	469 (37.05)	321 (44.15)	1	
		AG	583 (46.05)	311 (42.78)	**1.28 (1.05–1.56)**	**0.014**
		GG	214 (16.90)	95 (13.07)	**1.54 (1.16–2.04)**	**0.003**
		AG+GG	797 (62.95)	406 (55.85)	***1.35 (1.12*** **–** ***1.61)***	***0.0015***
IFNGR1	rs2234711	TT	395 (30.91)	266 (40.00)	1	
		CT	673 (52.66)	299 (44–96)	**1.52 (1.23–1.85)**	**<0.0001**
		CC	210 (16.43)	100 (15.04)	**1.41 (1.06–1.89)**	**0.017**
		CT+CC	883 (69.09)	399 (60.00)	***1.49 (1.22*** **–** ***1.82)***	***<0.0001***

1 Number of cases may differ due to missing data.

2 Two-sided X^2^ test for genotype distribution between the cases and controls, adjusted for age and gender.

No., number of subjects; OR, odds ratio; CI, confidence interval. Bold numbers indicate a statistical significance at 5% level.

Bold numbers in Italics indicate a statistical significance at 5% level after adjustment for multiple comparisons.

### Two SNPs were associated with CRC survival

**Figure 1:**
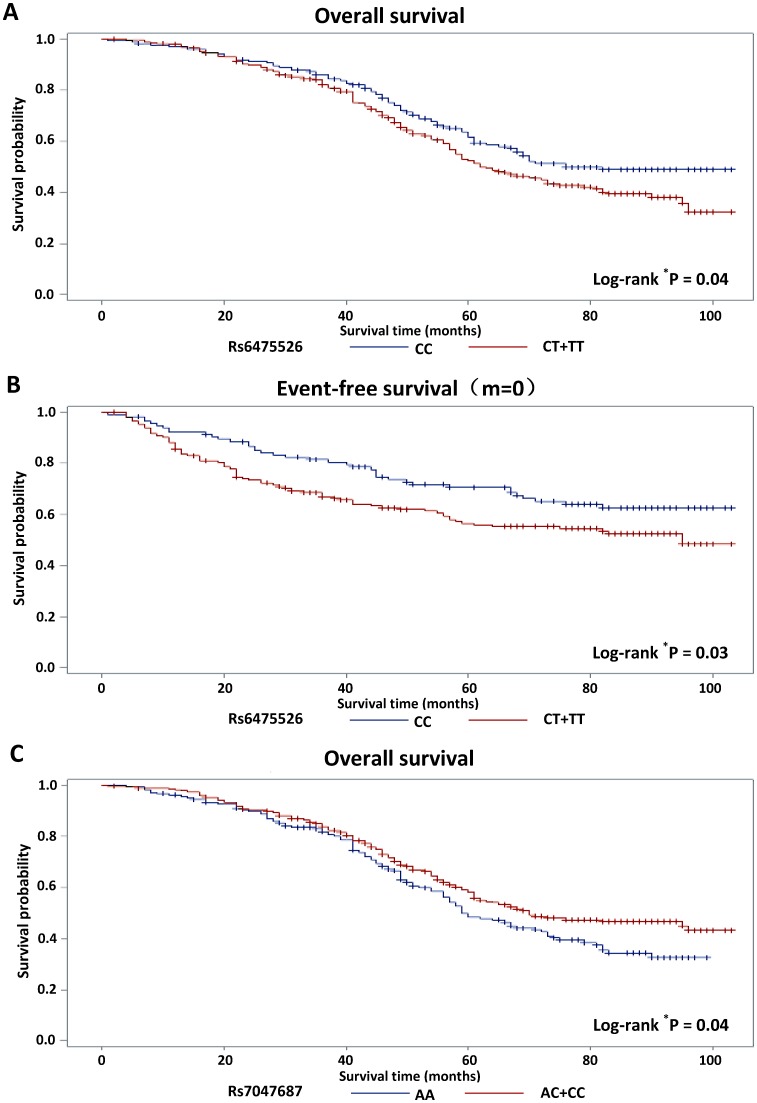
Kaplan-Meier analysis of survival according to genotypes of SNPs rs6475526, located 5′ to IFNA14, and capturing two IFN7 promoter SNPs and rs7047687 located in IFNA21 promoter. (A) Overall survival among all colorectal cancer patients (rs6475526, n = 465). (B) Event-free survival among patients without distant metastasis at diagnosis (rs6477526, n = 310). (C) Overall survival among all colorectal cancer patients (rs7047687, n = 464).

In the univariable analysis, the following parameters were associated with overall survival rate: gender, size or direct extent of the primary tumor (T), degree of spread to regional lymph nodes (N), presence of metastasis (M), TNM stage and tumor grade (Table S2). Interestingly, the SNP rs6475526, located about 2.2 kb 5′ of *IFNA14* and genotyped instead of the *IFNA7* promoter SNPs, and the *IFNA21* promoter SNP rs7047687, showed an association with overall survival among the 483 patients with follow-up data (HR 1.33, 95%CI 1.01–1.74 and HR 0.77, 95%CI 0.59–0.99, respectively) (Table 4, Table S3) and SNP rs6475526 also with event-free survival among patients without distant metastasis at the time of diagnosis (HR 1.51, 95%CI 1.03–2.21) (Table 4). Moreover, compared to the GG genotype carries, the AA carries of *IRF5* SNP rs11770859 had a better overall survival (HR 0.67, 95%CI 0.47–0.96). The Kaplan-Meier survival curves representing the overall and event-free survival rates of the patients according to their rs6475526 genotypes and the overall survival rates of the patients according to their rs7047687 genotypes are presented in Figure 1. The survival differences between the carriers of the different genotypes were statistically significant with log-rank p-values of 0.04, 0.03 and 0.04, respectively. The associations were strongest among stage 1 patients: the HR for overall survival was 4.04 (95%CI 1.13–14.53) for SNP rs6475526 and 0.29 (95%CI 0.10–0.83) for *IFNA21* SNP rs7047687; the HR for event-free survival was 3.78 (95%CI 1.27–11.67) for SNP rs6475526 (Table S4). However, these results should be taken with caution due to small number of patients who died (11/29 and 6/34 variant allele carriers of the stage 1 patients, respectively), and because the HRs among stage 2, 3 and 4 patients were similar to the ones for all 483 patients (Table S4). Moreover, no differences in overall survival between patients with grade 1+2 and grade 3+4 tumors or between patients without (M0) and with distant metastasis (M1) were observed. Stratified analysis according to tumor location showed that the worse overall survival of SNP rs6475526 was restricted to patients with rectal cancer (HR 2.10, 95%CI 1.31–3.36; colon cancer HR 1.01, 95%CI 0.72–1.42), the same tendency was observed also for event-free survival (rectal cancer HR 1.90, 95%CI 1.06–3.40; colon cancer HR 1.27, 95%CI 0.77–2.10) (Table S4).

**Table 4:** Association of rs7047687, rs6745526 and rs11770589 with overall and event-free survival of newly diagnosed colorectal cancer patients.

	No.^3^	No.^3^ died (%)	HR (95% CI)	*P* value	No.^3^	No.^3^ died (%)	HR (95% CI)	*P* value
Overall survival^1^					Adjusted for age, gender, diagnosis, TNM stage			
**rs7047687**								
**A/A**	181	101 (55.80)	1		159	88 (55.35)	1	
**A/C**	163	80 (49.08)	0.84 (0.62–1.12)	0.235	148	74 (50.00)	0.94 (0.69–1.28)	0.689
**C/C**	120	56 (46.67)	**0.69 (0.50–0.95)**	**0.025**	108	48 (44.44)	**0.65 (0.45–0.92)**	**0.016**
**dom**	283	136 (48.06)	**0.77 (0.59–1.00)**	**0.045**	256	122 (47.66)	0.79 (0.60–1.05)	0.101
								
**rs6475526**								
**C/C**	176	80 (45.45)	1		159	71 (44.65)	1	
**C/T**	224	125 (55.80)	**1.41 (1.07–1.87)**	**0.017**	203	112 (55.17)	**1.43 (1.06–1.93)**	**0.021**
**T/T**	65	28 (43.08)	1.05 (0.68–1.61)	0.834	55	25 (45.45)	1.26 (0.80–2.00)	0.324
**dom**	289	153 (52.94)	**1.33 (1.01–1.74)**	**0.041**	258	137	**1.38 (1.04–1.84)**	**0.029**
**rs11770589**								
**G/G**	142	79 (55.63)	1		128	69 (53.91)	1	
**A/G**	210	106 (50.48)	0.83 (0.62–1.11)	0.214	187	94 (50.27)	0.89 (0.65–1.21)	0.455
**A/A**	109	49 (44.95)	**0.67 (0.47–0.96)**	**0.028**	96	44 (45.83)	0.76 (0.52–1.11)	0.152
**dom**	319	155 (48.59)	0.77 (0.59–1.01)	0.062	283	138 (48.76)	0.85 (0.64–1.14)	0.269

**Event-free survival (M = 0)^2^**					**Adjusted for age, gender, diagnosis, TNM stage**			
	**No.^3^**	**No.^3^ event (%)**	**HR (95% CI)**	***P* value**	**No.^3^**	**No.^3^ event (%)**	**HR (95% CI)**	***P* value**
**A/A**	118	53 (44.92)	1		111	49 (44.14)	1	
**A/C**	112	44 (39.29)	0.82 (0.55–1.22)	0.323	106	42 (19.62)	0.82 (0.54–1.25)	0.354
**C/C**	83	33 (39.76)	0.77 (0.50–1.20)	0.250	77	28 (36.36)	0.72 (0.50–1.50)	0.168
**dom**	195	77 (39.49)	0.80 (0.56–1.13)	0.207	183	70 (38.25)	0.77 (0.54–1.12)	0.172
**rs6475526**								
**C/C**	115	39 (33.91)	1		107	34 (31.78)	1	
**C/T**	149	68 (45.64)	1.59 (1.07–2.36)	0.021	143	64 (44.76)	1.61 (1.06–2.46)	0.027
**T/T**	46	17 (36.96)	1.25 (0.71–2.22)	0.436	41	15 (36.59)	1.43 (0.77–2.66)	0.253
**dom**	195	85 (43.59)	1.51 (1.03–2.20)	0.034	184	79 (42.93)	1.55 (1.03–2.32)	0.036
**rs11770589**								
**G/G**	89	39 (43.82)	1		85	36 (42.35)	1	
**A/A**	81	32 (39.51)	0.86 (0.54–1.37)	0.52	76	30 (39.47)	0.95 (0.54–1.55)	0.845
**A/G**	140	58 (41.43)	0.93 (0.62–1.40)	0.73	131	53 (40.46)	0.96 (0.63–1.48)	0.855
**dom**	221	90 (40.72)	0.90 (0.62–1.32)	0.59	207	83 (40.10)	0.95 (0.64–1.40)	0.787

1 Overall survival was calculated for all patients diagnosed between 2003 and 2010 (n = 483).

2 Event-free survival was calculated for patients diagnosed between 2003 and 2010, who did not have distant metastasis at the time of diagnosis

(n = 325).

3 Number of cases may differ due to missing data.

No., number of patients; OR, odds ratio; CI, confidence interval; M = 0, no distant metastasis present Bold numbers indicate a statistical significance at 5% level.

In the multivariable analysis, the HRs for rs6475526 remained statistically significant after adjustment for age, gender, tumor location and TNM stage (overall survival HR 1.38, 95% CI 1.04–1.84; event-free survival HR 1.55, 95% CI 1.03–2.32) (Table 4).

## Discussion

IFN-signaling system may play a critical role in carcinogenesis of CRC by regulating immune responses during inflammation and it may additionally affect survival of CRC patients [8], [13]–[16], [20], [25]. In this genetic association study, we investigated the associations between 34 SNPs capturing 74 potentially functional SNPs in the IFN-signaling system genes and CRC risk and clinical outcome. Two SNPs located in the *IFNAR1* and *IFNGR1* genes exhibited an association with CRC risk. In the multivariable survival analysis, the SNP rs6475526, located about 2.2 kb of *IFNA14* and capturing two promoter SNPs in *IFNA7*, was associated with overall survival and also with event-free survival of non-metastatic CRC patients. These SNPs together with other common variants identified by the GWASs and the candidate gene studies may affect CRC risk and clinical outcome.

*IFNAR1* has recently been proposed as a novel candidate CRC tumor suppressor gene [21]. IFNAR1 has also been reported to play an important role in the development of early-onset CRC, suggesting a role in genetic predisposition [23]. Polymorphisms in *IFNAR1* have also been reported to be associated with susceptibility of multiple sclerosis, hepatocellular carcinoma and outcome of hepatitis B virus infection [32], [33]. In our study, the minor allele of rs2856968 in the intron of *IFNAR1* was associated with an increased risk of CRC. This polymorphism captured three promoter SNPs rs2843710, rs17875753 and rs17875752 with high LD (D′ = 1, r^2^ = 1). Rs2843710 is located in the binding site of protein polymerase (DNA directed), epsilon, catalytic subunit (POLE), which has been reported to be associated with colorectal carcinogenesis [26], [34]. Combined with the previously reported associations of *IFNAR1* with early-onset CRC and effects of *IFNAR1* on apoptosis and p53 signaling pathway in CRC cells [13], [21], [23], our data support the role of *IFNAR1* in CRC causation.

The IFNGR1 protein is a prerequisite to the initiation of IFNG signaling [35]. Reduced expression of IFNGR1 has been reported to be associated with clinicopathologic characteristics of esophageal cancer [36] and prognosis of ovarian cancer [37]. Published studies have reported that polymorphisms in *IFNGR1* are significantly associated with susceptibility of chronic hepatitis B virus infection, early gastric carcinoma, and rectal cancer [16], [38], [39]. In this study, we found the minor allele of rs2234711 in the promoter of *IFNGR1* to be associated with an increased risk of CRC. Rs2234711 has also been reported to be associated with the susceptibility of early gastric carcinoma, chronic hepatitis B virus infection and cerebral malaria [38]–[40]. A previous study indicated that rs2234711 may have functional effects on stimulating B cell lines, and C allele was associated with decreased *IFNGR1* gene activity, however, in a context-dependent manner [41]. Rs2234711 is located near an activating protein (AP)-2/AP-4 consensus binding site [42] and overexpression of AP-2α has been shown to reduce the expression of *IFNGR1* and to inhibit IFNG signaling [35]. Moreover, rs2234711 is located in the binding site of POLB, which has been associated with CRC [43], [44]. Together with evidences above, our finding suggested that the functional variant rs2234711 might have an effect on CRC causation through regulating the expression or function of *IFNGR1*.

Our study suggested an association of *IFNA* SNPs with clinical outcome of CRC. Based on our data, the SNP rs6475526 may be associated with overall and event-free survival of CRC patients. The associations remained statistically significant after adjustment for known prognostic markers, suggesting that rs6475526 is an independent prognostic marker. Rs6475526 (about 2.2 kb 5′ of IFNA14) is capturing two *IFNA7* promoter SNPs rs7045980 and rs7046208 with high LD (D′≥0.97, r^2^≥0.85). According to SNPinfo Web Server, all these SNPs are located nearby several transcription factor binding sites and may thus affect transcriptional activity. Previous studies have reported that IFNA-expressing tumor cells enhance generation and promote survival of tumor-specific cytotoxic T lymphocytes [45] and that IFNA improves the anti-proliferative effect of EGFR inhibitors in CRC cell lines [27], [28]. Our finding gives novel evidence of the role of IFNA in CRC progression and survival.

Compared to the previous study, which investigated the association of tagSNPs in *IFNG*, *IFNGR1*, *IFNGR2* and *IRF1-9* with the risk and survival of colon and rectal cancer [16], our study focused on potentially functional SNPs and covered in addition to the *IRFs*, *IFNG* and its receptors also other *IFNs* and their receptors. Six genes, *IFNG, IFNGR1, IFNGR2, IRF3, IRF5* and *IRF7*, were common in these two studies. In contrast to the previous study, which reported many associations both with colon and rectal cancer risk and survival, we observed only two associations with CRC risk and one association with overall and event-free survival. The only gene showing association in both studies was *IFNGR1*, however, the SNP rs2234711 which was associated with CRC risk in our study, was not covered by any tagSNP in the previous study. For the risk analysis, both studies were large [Slattery et. al. case/control, 1555/1956 (colon cancer), 754/959 (rectal cancer); we 1327/758 (CRC)]. There may be small differences in the origin of the study participants, with our study coming from a genetically quite uniform Czech population [22], while the recruitment area of the study by Slattery et. al. was Northern California and Utah, including also some 10–20% of Hispanic, Black and Asian participants. For the survival analysis, the studies had comparable follow-up time, but while Slattery et. al. had follow-up for all patients, we had it only for 483 patients, which decreased our power to detect small associations. However, this ensured that only newly diagnosed CRC cases (within one year of diagnosis before enrollment for this study) were included in our study, excluding a survival bias. For this subgroup, nearly complete clinical data were available, allowing evaluation of the SNPs as independent prognostic markers.

GWASs mainly describe only the most robust associations, which may be the reason that they have not reported any associations between CRC and interferon pathway genes. The tagSNP approach, used in the GWAS, is thought as a method with maximum SNP prediction accuracy, however, it does not cover all SNPs in the regulatory regions. A total of 74 SNPs in the regulatory and coding regions of the genes were covered by our study. However, due to sample size restrictions, we concentrated on SNPs with MAF ≥10% in Europeans and on SNPs located in the basic regulatory regions. It is possible that SNPs with a lower MAF or SNPs in still unknown regulatory regions of the studied genes, such as the enhancer and the silencer regions, might also modulate CRC susceptibility or survival.

In summary, our results, together with the previous study by Slattery et. al. suggest that genetic variation in the IFN signaling pathway genes plays a role in the etiology and survival of CRC. The strongest findings of our study included the associations of SNPs in *IFNAR1* and *IFNGR1* with susceptibility to CRC, and of SNPs in *IFNA7/IFNA14* with the survival of CRC patients. Validation of our findings and investigation of novel genetic variants in large, independent populations are encouraged.

## Supporting Information

Figure S1 **Haploview linkage disequilibrium (LD) pattern of the interferon alpha region on chromosome 9p21.3 showing pairwise LD values r^2^ between the SNPs.** Only SNPs with the minor allele frequency>10% in the Utah residents with Northern and Western European ancestry (CEU) from the CEPH collection in the 1000 genomes project are shown. Intensity of the gray color from white (r^2^ = 0) to black (r^2^ = 1) indicates the extent of LD. The two *IFNA7* promoter SNPs rs7045980 and rs7046208 captured by rs6475526 (2.2 kb of *IFNA14*) are surrounded by a red line.
(PNG)

Table S1 **Association of all evaluated SNPs with colorectal cancer susceptibility in the whole study population of 1327 cases and 758 controls.**
(DOC)

Table S2 **Univariable analysis of colorectal cancer survival and known prognostic factors.**
(DOC)

Table S3 **Association of all evaluated SNPs with colorectal cancer overall survival for all patients and event-free survival among patients without distant metastasis at the time of diagnosis**.
(DOC)

Table S4 **Stratified analysis of rs6475526, rs7047687 and rs11770589 for overall survival and rs6475526 for event-free survival among patients without distant metastasis at the time of diagnosis.**
(DOC)
