# Supplementary figures and images for: Single Nucleotide Polymorphisms within Interferon Signaling Pathway Genes Are Associated with Colorectal Cancer Susceptibility and Survival
Source: PLoS One. 2014 Oct 28;9(10):e111061. doi: 10.1371/journal.pone.0111061 (PMC4211713; doi:10.1371/journal.pone.0111061)

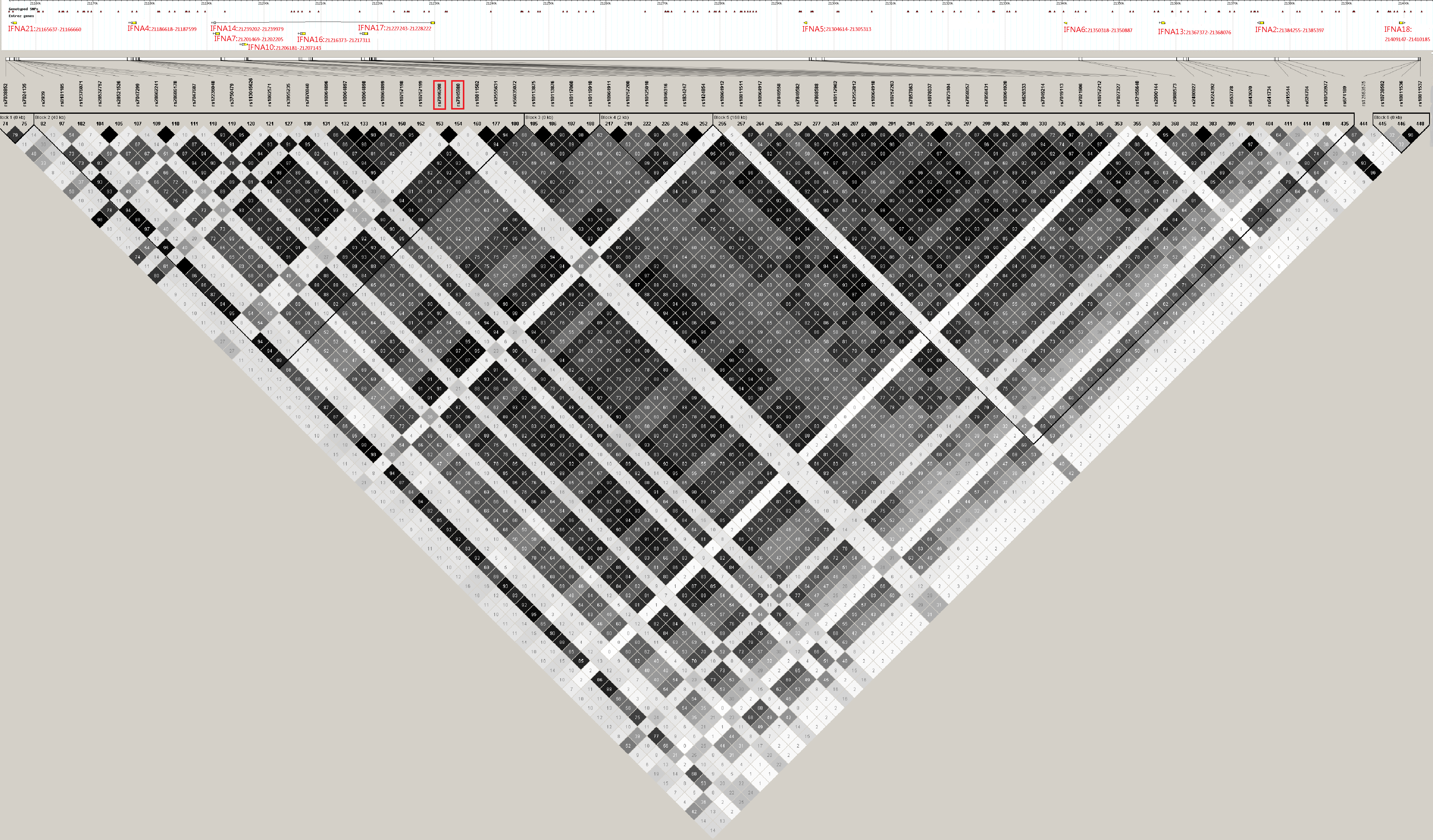

Supplement: Figure S1 — Haploview linkage disequilibrium (LD) pattern of the interferon alpha region on chromosome 9p21.3 showing pairwise LD values r2 between the SNPs. Only SNPs with the minor allele frequency>10% in the Utah residents with Northern and Western European ancestry (CEU) from the CEPH collection in the 1000 genomes project are shown. Intensity of the gray color from white (r2 = 0) to black (r2 = 1) indicates the extent of LD. The two IFNA7 promoter SNPs rs7045980 and rs7046208 captured by rs6475526 (2.2 kb of IFNA14) are surrounded by a red line. (PNG) [file pone.0111061.s001.png]
