# Supplementary material for: Single Nucleotide Polymorphisms within Interferon Signaling Pathway Genes Are Associated with Colorectal Cancer Susceptibility and Survival
Source: PLoS One. 2014 Oct 28;9(10):e111061. doi: 10.1371/journal.pone.0111061 (PMC4211713; doi:10.1371/journal.pone.0111061)
Supplement: Table S1 — Association of all evaluated SNPs with colorectal cancer susceptibility in the whole study population of 1327 cases and 758 controls. (DOC) [file pone.0111061.s002.doc]

**Table S1**. Association of all evaluated SNPs with colorectal cancer susceptibility in the whole study population of 1327 cases and 758 controls

| **SNP ID** | **Genotype** | **Case No.^1^(%)** | **Control No.^1^(%)** | **OR (95%CI)** | ***P* value** | **SNP ID** | **Genotype** | **Case No.^1^(%)** | **Control No.^1^(%)** | **OR (95%CI)** | ***P* value** |
| --- | --- | --- | --- | --- | --- | --- | --- | --- | --- | --- | --- |
| **rs33965070** | CC | 1060(82.23) | 640(86.37) | 1.00 |  | **rs10964859** | CC | 493(43.71) | 323(44.74) | 1.00 |  |
|  | CG | 229(17.77) | 101(13.63) | **1.37(1.06-1.75)** | **0.015** |  | CG | 494(43.79) | 303(41.97) | 1.06(0.88-1.3) | 0.519 |
|  | GG | 0(0) | 0(0) |  |  |  | GG | 141(12.50) | 96(13.3) | 0.96(0.71-1.3) | 0.798 |
|  | CG+GG | 229(17.77) | 101(13.63) | **1.37(1.06-1.75)** | **0.015** |  | CG+GG | 635(56.29) | 399(55.26) | 1.05(0.87-1.27) | 0.600 |
|  |  |  |  |  |  |  |  |  |  |  |  |
| **rs10120977** | AA | 801(61.38) | 440(62.23) | 1.00 |  | **rs2257167** | GG | 935(71.37) | 550(74.42) | 1.00 |  |
|  | AG | 434(33.26) | 238(33.66) | 1.01(0.82-1.22) | 0.987 |  | CG | 342(26.11) | 171(23.14) | 1.18(0.95-1.45) | 0.132 |
|  | GG | 70(5.36) | 29(4.1) | 1.33(0.85-2.08) | 0.217 |  | CC | 33(2.52) | 18(2.44) | 1.08(0.6-1.92) | 0.800 |
|  | AG+GG | 504(38.62) | 267(37.77) | 1.04(0.86-1.27) | 0.651 |  | CC+CG | 375(28.63) | 189(25.58) | 1.16(0.95-1.45) | 0.135 |
|  |  |  |  |  |  |  |  |  |  |  |  |
| **rs2383183** | TT | 1018(80.28) | 560(76.92) | 1.00 |  | **rs2834202** | AA | 715(56.08) | 448(62.22) | 1.00 |  |
|  | CT | 239(18.85) | 159(21.84) | 0.83(0.66-1.03) | 0.099 |  | AG | 481(37.73) | 230(31.94) | **1.32(1.08-1.59)** | **0.007** |
|  | CC | 11(0.87) | 9(1.24) | 0.67(0.28-1.64) | 0.380 |  | GG | 79(6.20) | 42(5.83) | 1.18(0.79-1.75) | 0.412 |
|  | CT+CC | 250(19.72) | 168(23.08) | 0.82(0.66-1.02) | 0.079 |  | AG+GG | 560(43.92) | 272(37.78) | **1.30(1.08-1.56)** | **0.007** |
|  |  |  |  |  |  |  |  |  |  |  |  |
| **rs12156640** | GG | 1006(79.15) | 562(78.6) | 1.00 |  | **rs2850015** | CC | 680(53.21) | 351(50.21) | 1.00 |  |
|  | AG | 253(19.91) | 149(20.84) | 0.95(0.76-1.19) | 0.649 |  | CT | 491(38.42) | 282(40.34) | 0.90(0.74-1.09) | 0.283 |
|  | AA | 12(0.94) | 4(.56) | 1.67(0.54-5.26) | 0.374 |  | TT | 107(8.37) | 66(9.44) | 0.84(0.6-1.16) | 0.294 |
|  | AG+GG | 265(20.85) | 153(21.4) | 0.96(0.77-1.2) | 0.739 |  | TC+CC | 598(46.79) | 630(90.13) | 0.88(0.74-1.06) | 0.207 |
|  |  |  |  |  |  |  |  |  |  |  |  |
| **rs6475526** | CC | 497(38.92) | 303(41.62) | 1.00 |  | **rs2856968** | AA | 469(37.05) | 321(44.15) | 1.00 |  |
|  | CT | 595(46.59) | 337(46.29) | 1.08(0.88-1.32) | 0.461 |  | AG | 583(46.05) | 311(42.78) | **1.28(1.05-1.56)** | **0.014** |
|  | TT | 185(14.49) | 88(12.09) | 1.56(0.96-2.5) | 0.095 |  | GG | 214(16.90) | 95(13.07) | 1.54(1.16-2.04) | 0.003 |
|  | TC+CC | 780(61.08) | 425(58.38) | 1.05(0.87-1.28) | 0.224 |  | AG+GG | 797(62.95) | 406(55.85) | **1.35(1.12-1.61)** | **0.002** |
|  |  |  |  |  |  |  |  |  |  |  |  |
| **rs10738592** | CC | 355(28.13) | 188(26.78) | 1.00 |  | **rs1131668** | GG | 591(45.29) | 330(45.45) | 1.00 |  |
|  | CT | 588(46.59) | 346(49.29) | 0.90(0.72-1.12) | 0.350 |  | AG | 574(43.98) | 318(43.8) | 1.01(0.83-1.22) | 0.936 |
|  | TT | 319(25.28) | 168(23.93) | 1.01(0.78-1.3) | 0.966 |  | AA | 140(10.73) | 78(10.74) | 1.01(0.74-1.37) | 0.989 |
|  | TC+CC | 907(71.87) | 514(73.22) | 0.94(0.76-1.16) | 0.573 |  | AG+AA | 714(54.71) | 396(54.55) | 1.01(0.87-1.2) | 0.956 |
|  |  |  |  |  |  |  |  |  |  |  |  |
| **rs10811536** | TT | 796(62.38) | 450(63.47) | 1.00 |  | **rs1327474** | AA | 389(30.06) | 222(29.68) |  |  |
|  | CT | 414(32.45) | 235(33.15) | 1.00(0.82-1.22) | 0.968 |  | AG | 667(51.55) | 383(51.2) | 0.99(0.81-1.22) | 0.954 |
|  | CC | 66(5.17) | 24(3.39) | 1.56(0.96-2.5) | 0.073 |  | GG | 238(18.39) | 143(19.12) | 0.95(0.73-1.23) | 0.702 |
|  | CT+CC | 480(37.62) | 259(36.53) | 1.05(0.87-1.28) | 0.584 |  | AG+GG | 905(69.94) | 526(70.32) | 0.98(0.81-1.19) | 0.845 |
|  |  |  |  |  |  |  |  |  |  |  |  |
| **rs12553575** | AA | 966(76.12) | 513(72.46) | 1.00 |  | **rs17181457** | CC | 1100(84.10) | 609(85.17) | 1.00 |  |
|  | AG | 277(21.83) | 186(26.27) | **0.79(0.64-1)** | **0.032** |  | CT | 201(15.37) | 101(14.13) | 0.91(0.71-1.18) | 0.45 |
|  | GG | 26(2.05) | 9(1.27) | 1.54(0.71-3.33) | 0.274 |  | TT | 7(0.54) | 5(0.70) | 1.29(0.41-4.08) | 0.67 |
|  | AG+GG | 303(23.88) | 195(27.54) | 0.83(0.68-1.03) | 0.093 |  | CT+TT | 208(15.90) | 106(14.83) | 0.92(0.72-1.12) | 0.53 |
|  |  |  |  |  |  |  |  |  |  |  |  |
| **rs641734** | CC | 900(69.39) | 473(64.35) | 1.00 |  | **rs2234711** | TT | 395(30.91) | 266(40.00) | 1.00 |  |
|  | CT | 358(27.6) | 239(32.52) | **0.79(0.65-0.96)** | **0.018** |  | CT | 673(52.66) | 299(44.96) | **1.52(1.23-1.85)** | **<0.001** |
|  | TT | 39(3.01) | 23(3.13) | 0.89(0.53-1.52) | 0.668 |  | CC | 210(16.43) | 100(15.04) | 1.41(1.06-1.89) | 0.017 |
|  | TC+CC | 397(30.61) | 262(35.65) | **0.80(0.66-0.97)** | **0.024** |  | CT+CC | 883(69.09) | 399(60.00) | **1.49(1.22-1.82)** | **<0.001** |
|  |  |  |  |  |  |  |  |  |  |  |  |
| **rs10964912** | AA | 727(56.36) | 423(59.58) | 1.00 |  | **rs1059293** | CC | 383(29.28) | 220(29.57) | 1.00 |  |
|  | AC | 490(37.98) | 259(36.48) | 1.10(0.91-1.33) | 0.328 |  | CT | 670(51.22) | 386(51.88) | 1.00(0.81-1.22) | 0.978 |
|  | CC | 73(5.66) | 28(3.94) | 1.52(0.96-2.38) | 0.071 |  | TT | 255(19.50) | 138(18.55) | 1.06(0.81-1.39) | 0.662 |
|  | AC+CC | 563(43.64) | 287(40.42) | 1.15(0.95-1.39) | 0.150 |  | CT+TT | 925(70.72) | 524(70.43) | 1.01(0.83-1.22) | 0.945 |
|  |  |  |  |  |  |  |  |  |  |  |  |
| **rs7873404** | TT | 751(60.08) | 429(58.37) | 1.00 |  | **rs17882748** | TT | 330(25.56) | 192(26.48) | 1.00 |  |
|  | CT | 451(36.08) | 269(36.6) | 0.96(0.79-1.16) | 0.659 |  | CT | 670(51.9) | 356(49.1) | 1.11(0.91-1.35) | 0.418 |
|  | CC | 48(3.84) | 37(5.03) | 0.74(0.47-1.15) | 0.187 |  | CC | 291(22.54) | 177(24.41) | 0.96(0.75-1.21) | 0.736 |
|  | CT+CC | 499(39.92) | 306(41.63) | 0.93(0.78-1.12) | 0.453 |  | CT+CC | 961(74.44) | 533(73.52) | 1.04(0.85-1.28) | 0.698 |
|  |  |  |  |  |  |  |  |  |  |  |  |
| **rs12376071** | AA | 575(44.78) | 350(47.68) | 1.00 |  | **rs9808753** | AA | 1029(78.73) | 574(77.67) | 1.00 |  |
|  | AG | 576(44.86) | 320(43.6) | 1.08(0.91-1.33) | 0.348 |  | AG | 262(20.05) | 152(20.57) | 0.96(0.77-1.2) | 0.732 |
|  | GG | 133(10.36) | 64(8.72) | 1.27(0.91-1.75) | 0.158 |  | GG | 16(1.22) | 13(1.76) | 0.68(0.33-1.45) | 0.319 |
|  | AG+GG | 709(55.22) | 384(52.32) | 1.14(0.94-1.37) | 0.186 |  | AG+GG | 278(21.27) | 165(22.33) | 0.95(0.79-1.15) | 0.502 |
|  |  |  |  |  |  |  |  |  |  |  |  |
| **rs2939** | TT | 882(67.9) | 466(62.38) | 1.00 |  | **rs2304204** | CC | 644(49.12) | 369(49.4) | 1.00 |  |
|  | CT | 383(29.48) | 257(34.40) | **0.79(0.65-0.95)** | **0.016** |  | CG | 556(42.41) | 304(40.7) | 0.98(0.81-1.19) | 0.810 |
|  | CC | 34(2.62) | 24(3.21) | 0.75(0.44-1.28) | 0.288 |  | GG | 111(8.47) | 74(9.91) | 0.86(0.58-1.28) | 0.460 |
|  | CT+CC | 417(32.1) | 281(37.62) | **0.79(0.65-0.95)** | **0.012** |  | CG+GG | 667(50.88) | 378(50.6) | 1.02(0.85-1.22) | 0.829 |
|  |  |  |  |  |  |  |  |  |  |  |  |
| **rs7047687** | AA | 460(36.28) | 258(36.65) | 1.00 |  | **rs11770589** | GG | 372(29.15) | 208(28.45) | 1.00 |  |
|  | AC | 445(35.09) | 206(29.26) | 1.20(0.97-1.52) | 0.094 |  | AG | 601(47.10) | 350(47.88) | 0.96(0.78-1.19) | 0.711 |
|  | CC | 363(28.63) | 240(34.09) | 0.85(0.68-1.06) | 0.149 |  | AA | 303(23.75) | 173(23.67) | 0.98(0.76-1.27) | 0.871 |
|  | AC+CC | 808(63.72) | 446(63.35) | 1.01(0.83-1.23) | 0.931 |  | AG+GG | 904(70.85) | 523(71.55) | 0.97(0.79-1.19) | 0.785 |
|  |  |  |  |  |  |  |  |  |  |  |  |
| **rs1424855** | CC | 573(44.25) | 310(41.95) | 1.00 |  | **rs1874327** | TT | 555(43.74) | 308(44.) | 1.00 |  |
|  | CG | 573(44.25) | 337(45.6) | 0.92(0.76-1.12) | 0.396 |  | AT | 556(43.81) | 283(40.43) | 1.09(0.89-1.33) | 0.369 |
|  | GG | 149(11.51) | 92(12.45) | 0.88(0.65-1.18) | 0.379 |  | AA | 158(12.45) | 109(15.57) | 0.81(0.61-1.06) | 0.129 |
|  | CG+GG | 722(55.75) | 429(58.05) | 0.91(0.76-1.1) | 0.329 |  | AT+AA | 714(56.26) | 392(56.) | 1.01(0.84-1.22) | 0.924 |
|  |  |  |  |  |  |  |  |  |  |  |  |
| **rs700782** | GG | 743(57.15) | 467(62.18) | 1.00 |  | **rs2070197** | TT | 1010(77.39) | 588(79.46) | 1.00 |  |
|  | AG | 484(37.23) | 252(33.56) | 1.20(1.00-1.47) | 0.054 |  | CT | 269(20.61) | 146(19.73) | 1.08(0.85-1.35) | 0.542 |
|  | AA | 73(5.62) | 32(4.26) | 1.43(0.93-2.22) | 0.102 |  | CC | 26(1.99) | 6(.81) | **2.50(1.03-6.25)** | **0.042** |
|  | AG+AA | 557(42.85) | 284(37.82) | **1.23(1.03-1.49)** | **0.023** |  | CT+CC | 295(22.61) | 152(20.54) | 1.12(0.9-1.41) | 0.290 |
|  |  |  |  |  |  |  |  |  |  |  |  |
| **rs10757189** | GG | 669(53.05) | 407(58.73) | 1.00 |  | **rs1061502** | TT | 747(59.66) | 420(60.78) | 1.00 |  |
|  | AG | 487(38.62) | 228(32.9) | 1.08(0.88-1.3) | 0.477 |  | CT | 416(33.23) | 223(32.27) | 1.05(0.85-1.28) | 0.644 |
|  | AA | 105(8.33) | 58(8.37) | 1.10(0.78-1.56) | 0.572 |  | CC | 89(7.11) | 48(6.95) | 1.04(0.72-1.52) | 0.826 |
|  | AG+AA | 592(46.95) | 286(41.27) | 1.08(0.89-1.28) | 0.451 |  | CT+CC | 505(40.34) | 271(39.22) | 1.05(0.87-1.27) | 0.623 |

^1^Number of cases may differ due to missing data. No., number of subjects; OR, odds ratio; CI, confidence interval. Bold numbers indicate a statistical significance at 5% level.
