# Supplementary material for: Single Nucleotide Polymorphisms within Interferon Signaling Pathway Genes Are Associated with Colorectal Cancer Susceptibility and Survival
Source: PLoS One. 2014 Oct 28;9(10):e111061. doi: 10.1371/journal.pone.0111061 (PMC4211713; doi:10.1371/journal.pone.0111061)
Supplement: Table S2 — Univariable analysis of colorectal cancer survival and known prognostic factors. (DOC) [file pone.0111061.s003.doc]

**Table S2.** Univariable analysis of colorectal cancer survival and known prognostic factors

| **Characteristics** | **No.^1^** | **No.^1^ died (%)** | **HR(95%CI)** | ***P* value** |
| --- | --- | --- | --- | --- |
| Age at diagnosis |  |  |  |  |
| <65 | 243 | 117(48.15) |  |  |
| ≥65 | 240 | 128(53.33) | 1.22(0.95-1.57) | 0.1208 |
| Gender |  |  |  |  |
| Female | 180 | 73(40.56) |  |  |
| Male | 303 | 172(56.77) | 1.668(1.27-2.21) | **0.0003** |
| Diagnosis |  |  |  |  |
| Colon | 298 | 149(50.00) |  |  |
| Rectum | 185 | 96(51.89) | 0.927(0.72-1.2) | 0.5607 |
| Grade |  |  |  |  |
| 1, 2 | 309 | 143(46.28) |  |  |
| 3, 4 | 105 | 56(53.33) | 1.673(1.22-2.27) | **0.0012** |
| T |  |  |  |  |
| T1, T2 | 88 | 26(29.55) |  |  |
| T3, T4 | 351 | 184(52.42) | 2.463(1.66-3.8) | **<.0001** |
| N |  |  |  |  |
| N0 | 216 | 78(36.11) |  |  |
| N1, N2 | 191 | 112(58.64) | 2.111(1.58-2.83) | **<.0001** |
| M |  |  |  |  |
| M0 | 325 | 116(35.69) |  |  |
| M1 | 126 | 110(87.30) | 4.544(3.46-5.96) | **<.0001** |
| TNM Stage |  |  |  |  |
| Stage I | 55 | 15(27.27) |  |  |
| Stage II | 128 | 37(28.91) | 1.203(0.67-2.26) | 0.5453 |
| Stage III | 123 | 55(44.72) | 2.173(1.26-3.99) | **0.0078** |
| Stage IV | 126 | 110(87.30) | 7.141(4.27-12.84) | **<.0001** |

^1^Number of cases may differ due to missing data.

No., number of patients; T, size or direct extent of the primary tumor; N, degree of spread to regional lymph nodes; M, presence of metastasis; HR, hazard ratio; CI, confidence interval

Bold numbers indicate a statistical significance at 5% level.
