# Supplementary material for: Single Nucleotide Polymorphisms within Interferon Signaling Pathway Genes Are Associated with Colorectal Cancer Susceptibility and Survival
Source: PLoS One. 2014 Oct 28;9(10):e111061. doi: 10.1371/journal.pone.0111061 (PMC4211713; doi:10.1371/journal.pone.0111061)
Supplement: Table S3 — Association of all evaluated SNPs with colorectal cancer overall survival for all patients and event-free survival among patients without distant metastasis at the time of diagnosis. (DOC) [file pone.0111061.s004.doc]

**Table S3**. Association of all evaluated SNPs with colorectal cancer overall survival for all patients and event-free survival among patients without distant metastasis at the time of diagnosis.

| **SNP ID** | **Overall survival** | | |  |  |  | **Event-free survival (M=0)** | | |  |
| --- | --- | --- | --- | --- | --- | --- | --- | --- | --- | --- |
|  |  | **No.^1^** | **No.^1^ died (%)** | **HR(95%CI)** | ***P* value** |  | **No.^1^** | **No.^1^ event (%)** | **HR(95%CI)** | ***P* value** |
| **rs33965070** | **C/C** | 386 | 189(48.96) | 1 |  |  | 260 | 102(39.23) | 1 |  |
|  | **C/G** | 84 | 47(55.95) | 1.04(0.75-1.43) | 0.82 |  | 56 | 25(44.64) | 1.15(0.74-1.77) | 0.55 |
|  | **G/G** | 0 | 0 |  |  |  | 0 | 0 |  |  |
|  | **dom** | 84 | 47(55.95) | 1.04(0.75-1.43) | 0.82 |  | 56 | 25(44.64) | 1.15(0.74-1.77) | 0.55 |
|  |  |  |  |  |  |  |  |  |  |  |
| **rs10120977** | **A/A** | 298 | 151(50.67) | 1 |  |  | 203 | 89(43.84) | 1 |  |
|  | **A/G** | 148 | 77(52.03) | 1.03(0.78-1.35) | 0.86 |  | 93 | 30(32.26) | 0.71(0.47-1.07) | 0.10 |
|  | **G/G** | 26 | 9(34.62) | 0.71(0.36-1.38) | 0.31 |  | 19 | 6(31.58) | 0.72(0.31-1.64) | 0.43 |
|  | **dom** | 174 | 86(49.43) | 0.98(0.75-1.28) | 0.87 |  | 112 | 36(32.14) | 0.71(0.48-1.05) | 0.08 |
|  |  |  |  |  |  |  |  |  |  |  |
| **rs2383183** | **T/T** | 365 | 180(49.32) | 1 |  |  | 256 | 101(39.45) | 1 |  |
|  | **C/T** | 92 | 50(54.35) | 1.22(0.89-1.66) | 0.22 |  | 2 | 0(0) | 0 | 0.98 |
|  | **C/C** | 4 | 1(25.00) | 0.35(0.05-2.49) | 0.29 |  | 52 | 24(46.15) | 1.1(0.71-1.72) | 0.67 |
|  | **dom** | 96 | 51(53.13) | 1.16(0.85-1.58) | 0.35 |  | 54 | 24(44.44) | 1.05(0.67-1.64) | 0.84 |
|  |  |  |  |  |  |  |  |  |  |  |
| **rs12156640** | **G/G** | 367 | 186(50.68) | 1 |  |  | 237 | 90(37.97) | 1 |  |
|  | **A/G** | 99 | 51(51.52) | 1.11(0.82-1.52) | 0.50 |  | 4 | 2(50.00) | 1.57(0.39-6.36) | 0.53 |
|  | **A/A** | 5 | 1(20.00) | 0.3(0.04-2.13) | 0.23 |  | 76 | 37(48.68) | 1.43(0.98-2.10) | 0.07 |
|  | **dom** | 104 | 52(50.00) | 1.06(0.78-1.44) | 0.72 |  | 80 | 39(48.75) | 1.44(0.99-2.09) | 0.06 |
|  |  |  |  |  |  |  |  |  |  |  |
| **rs6475526** | **C/C** | 176 | 80(45.45) | 1 |  |  | 115 | 39(33.91) | 1 |  |
|  | **C/T** | 224 | 125(55.8) | **1.41(1.07-1.87)** | **0.02** |  | 149 | 68(45.64) | **1.59(1.07-2.36)** | **0.02** |
|  | **T/T** | 65 | 28(43.08) | 1.05(0.68-1.61) | 0.83 |  | 46 | 17(36.96) | 1.25(0.71-2.22) | 0.44 |
|  | **dom** | 289 | 153(52.94) | **1.33(1.01-1.74)** | **0.04** |  | 195 | 85(43.59) | **1.51(1.03-2.2)** | **0.03** |
|  |  |  |  |  |  |  |  |  |  |  |
| **rs10738592** | **T/T** | 120 | 63(52.50) | 1 |  |  | 81 | 34(41.98) | 1 |  |
|  | **C/T** | 228 | 114(50.00) | 0.98(0.72-1.33) | 0.88 |  | 72 | 26(36.11) | 0.81(0.48-1.34) | 0.41 |
|  | **C/C** | 113 | 56(49.56) | 0.97(0.68-1.39) | 0.87 |  | 156 | 66(42.31) | 1(0.66-1.52) | 0.99 |
|  | **dom** | 348 | 177(50.86) | 0.97(0.73-1.3) | 0.86 |  | 128 | 92(71.88) | 0.94(0.63-1.39) | 0.75 |
|  |  |  |  |  |  |  |  |  |  |  |
| **rs10811536** | **T/T** | 295 | 147(49.83) | 1 |  |  | 199 | 86(43.22) | 1 |  |
|  | **C/T** | 140 | 76(54.29) | 1.06(0.8-1.39) | 0.71 |  | 19 | 7(36.84) | 0.85(0.39-1.83) | 0.67 |
|  | **C/C** | 27 | 11(40.74) | 0.79(0.43-1.45) | 0.44 |  | 91 | 33(36.26) | 0.82(0.55-1.23) | 0.33 |
|  | **dom** | 167 | 87(52.1) | 1.01(0.78-1.32) | 0.94 |  | 110 | 40(36.36) | 0.83(0.57-1.2) | 0.31 |
|  |  |  |  |  |  |  |  |  |  |  |
| **rs12553575** | **A/A** | 350 | 173(49.43) | 1 |  |  | 243 | 97(39.92) | 1 |  |
|  | **A/G** | 104 | 59(56.73) | 1.17(0.87-1.58) | 0.29 |  | 64 | 32(50.00) | 1.21(0.81-1.81) | 0.34 |
|  | **G/G** | 13 | 3(23.08) | 0.41(0.13-1.28) | 0.13 |  | 10 | 1(10.00) | 0.2(0.03-1.46) | 0.11 |
|  | **dom** | 117 | 62(52.99) | 1.08(0.8-1.44) | 0.62 |  | 74 | 33(44.59) | 1.06(0.71-1.57) | 0.79 |
|  |  |  |  |  |  |  |  |  |  |  |
| **rs641734** | **C/C** | 312 | 157(50.32) | 1 |  |  | 213 | 86(40.38) | 1 |  |
|  | **C/T** | 139 | 72(51.8) | 1.02(0.77-1.35) | 0.89 |  | 90 | 40(44.44) | 1.03(0.71-1.5) | 0.89 |
|  | **T/T** | 20 | 8(40.00) | 0.78(0.38-1.58) | 0.49 |  | 13 | 2(15.38) | 0.31(0.08-1.25) | 0.10 |
|  | **dom** | 159 | 80(50.31) | 0.99(0.76-1.29) | 0.93 |  | 103 | 42(40.78) | 0.93(0.64-1.34) | 0.68 |
|  |  |  |  |  |  |  |  |  |  |  |
| **rs10964912** | **A/A** | 269 | 132(49.07) | 1 |  |  | 184 | 77(41.85) | 1 |  |
|  | **A/C** | 179 | 96(53.63) | 1.16(0.89-1.51) | 0.27 |  | 120 | 48(40.00) | 0.97(0.68-1.39) | 0.88 |
|  | **C/C** | 25 | 12(48.00) | 0.96(0.53-1.74) | 0.90 |  | 15 | 6(40.00) | 0.99(0.43-2.28) | 0.99 |
|  | **dom** | 204 | 108(52.94) | 1.13(0.88-1.46) | 0.34 |  | 135 | 54(40.00) | 0.97(0.69-1.38) | 0.88 |
|  |  |  |  |  |  |  |  |  |  |  |
| **rs7873404** | **T/T** | 262 | 130(49.62) | 1 |  |  | 178 | 70(39.33) | 1 |  |
|  | **C/T** | 179 | 92(51.4) | 1.05(0.81-1.37) | 0.72 |  | 8 | 3(37.50) | 0.86(0.27-2.74) | 0.80 |
|  | **C/C** | 17 | 11(64.71) | 1.29(0.7-2.39) | 0.41 |  | 120 | 53(44.17) | 1.08(0.76-1.54) | 0.67 |
|  | **dom** | 196 | 103(52.55) | 1.07(0.83-1.39) | 0.59 |  | 128 | 56(43.75) | 1.07(0.75-1.51) | 0.72 |
|  |  |  |  |  |  |  |  |  |  |  |
| **rs12376071** | **A/A** | 205 | 104(50.73) |  |  |  | 135 | 58(42.96) | 1 |  |
|  | **A/G** | 209 | 108(51.67) | 1.14(0.87-1.49) | 0.35 |  | 142 | 55(38.73) | 0.95(0.65-1.37) | 0.77 |
|  | **G/G** | 51 | 26(50.98) | 1.21(0.79-1.86) | 0.38 |  | 32 | 13(40.63) | 1.13(0.62-2.07) | 0.68 |
|  | **dom** | 260 | 134(51.54) | 1.15(0.89-1.49) | 0.28 |  | 174 | 68(39.08) | 0.98(0.69-1.39) | 0.90 |
|  |  |  |  |  |  |  |  |  |  |  |
| **rs2939** | **T/T** | 313 | 157(50.16) | 1 |  |  | 214 | 87(40.65) | 1 |  |
|  | **C/T** | 149 | 75(50.34) | 0.98(0.75-1.29) | 0.90 |  | 8 | 1(12.50) | 0.25(0.04-1.8) | 0.17 |
|  | **C/C** | 15 | 7(46.67) | 0.87(0.41-1.85) | 0.71 |  | 100 | 42(42.00) | 0.97(0.67-1.4) | 0.86 |
|  | **dom** | 164 | 82(50.00) | 0.97(0.74-1.27) | 0.83 |  | 108 | 43(39.81) | 0.91(0.63-1.31) | 0.60 |
|  |  |  |  |  |  |  |  |  |  |  |
| **rs7047687** | **A/A** | 181 | 101(55.80) | 1 |  |  | 118 | 53(44.92) | 1 |  |
|  | **A/C** | 163 | 80(49.08) | 0.84(0.62-1.12) | 0.24 |  | 112 | 44(39.29) | 0.82(0.55-1.22) | 0.32 |
|  | **C/C** | 120 | 56(46.67) | **0.69(0.5-0.95)** | **0.02** |  | 83 | 33(39.76) | 0.77(0.5-1.2) | 0.25 |
|  | **dom** | 283 | 136(48.06) | **0.77(0.59-1.00)** | **0.05** |  | 195 | 77(39.49) | 0.8(0.56-1.13) | 0.21 |
|  |  |  |  |  |  |  |  |  |  |  |
| **rs1424855** | **C/C** | 196 | 101(51.53) | 1 |  |  | 128 | 53(41.41) | 1 |  |
|  | **C/G** | 223 | 106(47.53) | 0.95(0.72-1.25) | 0.71 |  | 158 | 62(39.24) | 1(0.69-1.44) | 0.98 |
|  | **G/G** | 53 | 30(56.60) | 1.14(0.76-1.71) | 0.53 |  | 32 | 14(43.75) | 1.05(0.58-1.89) | 0.87 |
|  | **dom** | 276 | 136(49.28) | 0.99(0.76-1.28) | 0.91 |  | 190 | 76(40.00) | 1(0.71-1.43) | 0.98 |
|  |  |  |  |  |  |  |  |  |  |  |
| **rs700782** | **G/G** | 276 | 142(51.45) | 1 |  |  | 186 | 77(41.40) | 1 |  |
|  | **A/G** | 177 | 90(50.85) | 0.89(0.69-1.17) | 0.41 |  | 16 | 3(18.75) | 0.4(0.13-1.27) | 0.12 |
|  | **A/A** | 24 | 10(41.67) | 0.73(0.38-1.39) | 0.34 |  | 119 | 52(43.70) | 1.07(0.76-1.53) | 0.69 |
|  | **dom** | 201 | 100(49.75) | 0.88(0.68-1.13) | 0.31 |  | 135 | 55(40.74) | 0.98(0.7-1.39) | 0.92 |
|  |  |  |  |  |  |  |  |  |  |  |
| **rs10757189** | **G/G** | 232 | 115(49.57) | 1 |  |  | 156 | 62(39.74) | 1 |  |
|  | **A/G** | 187 | 102(54.55) | 1.14(0.87-1.48) | 0.35 |  | 30 | 11(36.67) | 0.91(0.48-1.73) | 0.77 |
|  | **A/A** | 39 | 17(43.59) | 0.83(0.5-1.38) | 0.46 |  | 120 | 54(45.00) | 1.18(0.82-1.7) | 0.38 |
|  | **dom** | 226 | 119(52.65) | 1.08(0.84-1.4) | 0.56 |  | 150 | 65(43.33) | 1.12(0.79-1.59) | 0.52 |
|  |  |  |  |  |  |  |  |  |  |  |
| **rs10964859** | **C/C** | 170 | 89(52.35) | 1 |  |  | 116 | 50(43.10) | 1 |  |
|  | **C/G** | 174 | 85(48.85) | 0.91(0.67-1.22) | 0.51 |  | 113 | 47(41.59) | 0.93(0.63-1.39) | 0.73 |
|  | **G/G** | 38 | 20(52.63) | 0.96(0.59-1.55) | 0.85 |  | 28 | 12(42.86) | 1.03(0.55-1.93) | 0.93 |
|  | **dom** | 212 | 105(49.53) | 0.91(0.69-1.21) | 0.53 |  | 141 | 59(41.84) | 0.95(0.65-1.39) | 0.79 |
|  |  |  |  |  |  |  |  |  |  |  |
| **rs2257167** | **G/G** | 323 | 159(49.23) | 1 |  |  | 219 | 85(38.81) | 1 |  |
|  | **C/G** | 141 | 72(51.06) | 1.14(0.86-1.5) | 0.36 |  | 5 | 1(20.00) | 0.44(0.06-3.15) | 0.41 |
|  | **C/C** | 13 | 9(69.23) | 1.71(0.87-3.35) | 0.12 |  | 99 | 45(45.45) | 1.24(0.87-1.79) | 0.24 |
|  | **dom** | 154 | 81(52.6) | 1.18(0.91-1.55) | 0.22 |  | 104 | 46(44.23) | 1.2(0.84-1.71) | 0.33 |
|  |  |  |  |  |  |  |  |  |  |  |
| **rs2834202** | **A/A** | 256 | 138(53.91) | 1 |  |  | 165 | 73(44.24) | 1 |  |
|  | **A/G** | 180 | 86(47.78) | 0.93(0.71-1.21) | 0.57 |  | 125 | 50(40.00) | 0.96(0.67-1.37) | 0.80 |
|  | **G/G** | 24 | 11(45.83) | 0.86(0.47-1.59) | 0.64 |  | 18 | 6(33.33) | 0.79(0.34-1.81) | 0.57 |
|  | **dom** | 204 | 97(47.55) | 0.92(0.71-1.19) | 0.52 |  | 143 | 56(39.16) | 0.93(0.66-1.32) | 0.70 |
|  |  |  |  |  |  |  |  |  |  |  |
| **rs2850015** | **C/C** | 264 | 138(52.27) | 1 |  |  | 181 | 78(43.09) | 1 |  |
|  | **C/T** | 163 | 80(49.08) | 0.93(0.7-1.22) | 0.59 |  | 103 | 36(34.95) | 0.79(0.54-1.18) | 0.25 |
|  | **T/T** | 42 | 18(42.86) | 0.66(0.4-1.07) | 0.09 |  | 30 | 11(36.67) | 0.74(0.39-1.38) | 0.34 |
|  | **dom** | 205 | 98(47.80) | 0.86(0.67-1.12) | 0.26 |  | 133 | 47(35.34) | 0.78(0.54-1.12) | 0.18 |
|  |  |  |  |  |  |  |  |  |  |  |
| **rs2856968** | **A/A** | 156 | 75(48.08) | 1 |  |  | 101 | 39(38.61) | 1 |  |
|  | **A/G** | 220 | 120(54.55) | 1.23(0.92-1.64) | 0.16 |  | 143 | 64(44.76) | 1.27(0.85-1.89) | 0.24 |
|  | **G/G** | 84 | 35(41.67) | 0.9(0.6-1.34) | 0.59 |  | 64 | 20(31.25) | 0.82(0.48-1.41) | 0.47 |
|  | **dom** | 304 | 155(50.99) | 1.14(0.86-1.5) | 0.37 |  | 207 | 84(40.58) | 1.12(0.77-1.64) | 0.55 |
|  |  |  |  |  |  |  |  |  |  |  |
| **rs1131668** | **G/G** | 216 | 103(47.69) | 1 |  |  | 137 | 48(35.04) | 1 |  |
|  | **A/G** | 213 | 110(51.64) | 0.99(0.76-1.3) | 0.95 |  | 30 | 13(43.33) | 1.31(0.71-2.42) | 0.39 |
|  | **A/A** | 49 | 29(59.18) | 1.42(0.94-2.15) | 0.09 |  | 154 | 70(45.45) | 1.28(0.89-1.85) | 0.19 |
|  | **dom** | 262 | 139(53.05) | 1.06(0.82-1.37) | 0.66 |  | 184 | 83(45.11) | 1.29(0.9-1.83) | 0.17 |
|  |  |  |  |  |  |  |  |  |  |  |
| **rs1327474** | **A/A** | 139 | 66(47.48) | 1 |  |  | 103 | 45(43.69) | 1 |  |
|  | **A/G** | 250 | 133(53.20) | 1.16(0.86-1.56) | 0.32 |  | 159 | 63(39.62) | 0.85(0.58-1.25) | 0.40 |
|  | **G/G** | 88 | 43(48.86) | 1.07(0.73-1.57) | 0.73 |  | 58 | 23(39.66) | 0.82(0.49-1.35) | 0.42 |
|  | **dom** | 338 | 176(52.07) | 1.14(0.86-1.51) | 0.37 |  | 217 | 86(39.63) | 0.84(0.59-1.2) | 0.34 |
|  |  |  |  |  |  |  |  |  |  |  |
| **rs17181457** | **C/C** | 396 | 203(51.26) | 1 |  |  | 262 | 109(41.60) | 1 |  |
|  | **C/T** | 79 | 39(49.37) | 1.04(0.74-1.47) | 0.81 |  | 56 | 22(39.29) | 1.03(0.65-1.64) | 0.89 |
|  | **T/T** | 4 | 2(50.00) | 0.95(0.24-3.81) | 0.94 |  | 3 | 1(33.33) | 0.7(0.1-4.97) | 0.72 |
|  | **dom** | 83 | 41(49.40) | 1.04(0.74-1.45) | 0.82 |  | 59 | 23(38.98) | 1.01(0.65-1.59) | 0.96 |
|  |  |  |  |  |  |  |  |  |  |  |
| **rs2234711** | **T/T** | 144 | 74(51.39) | 1 |  |  | 90 | 36(40.00) | 1 |  |
|  | **C/T** | 246 | 121(49.19) | 0.87(0.65-1.16) | 0.33 |  | 52 | 24(46.15) | 1.41(0.84-2.37) | 0.19 |
|  | **C/C** | 73 | 37(50.68) | 1(0.67-1.48) | 0.99 |  | 167 | 64(38.32) | 1(0.66-1.5) | 0.98 |
|  | **dom** | 319 | 158(49.53) | 0.89(0.68-1.18) | 0.42 |  | 219 | 88(40.18) | 1.08(0.73-1.6) | 0.69 |
|  |  |  |  |  |  |  |  |  |  |  |
| **rs1059293** | **C/C** | 129 | 69(53.49) |  |  |  | 89 | 41(46.07) | 1 |  |
|  | **C/T** | 256 | 126(49.22) | 0.93(0.69-1.25) | 0.63 |  | 165 | 62(37.58) | 0.77(0.52-1.14) | 0.20 |
|  | **T/T** | 94 | 47(50.00) | 0.86(0.6-1.25) | 0.44 |  | 68 | 28(41.18) | 0.78(0.48-1.26) | 0.31 |
|  | **dom** | 350 | 173(49.43) | 0.93(0.7-1.24) | 0.62 |  | 233 | 90(38.63) | 0.77(0.54-1.12) | 0.17 |
|  |  |  |  |  |  |  |  |  |  |  |
| **rs17882748** | **T/T** | 121 | 64(52.89) | 1 |  |  | 80 | 29(36.25) | 1 |  |
|  | **C/T** | 243 | 123(50.62) | 0.94(0.65-1.36) | 0.74 |  | 74 | 27(36.49) | 1.05(0.62-1.78) | 0.85 |
|  | **C/C** | 106 | 52(49.06) | 0.92(0.68-1.24) | 0.57 |  | 161 | 73(45.34) | 1.32(0.86-2.03) | 0.20 |
|  | **dom** | 349 | 175(50.14) | 0.92(0.69-1.23) | 0.59 |  | 235 | 100(42.55) | 1.24(0.82-1.87) | 0.32 |
|  |  |  |  |  |  |  |  |  |  |  |
| **rs9808753** | **A/A** | 384 | 194(50.52) | 1 |  |  | 261 | 106(40.61) | 1 |  |
|  | **A/G** | 88 | 47(53.41) | 1.08(0.79-1.49) | 0.62 |  | 57 | 26(45.61) | 1.08(0.7-1.65) | 0.74 |
|  | **G/G** | 7 | 3(42.86) | 0.64(0.21-2.01) | 0.44 |  | 4 | 1(25.00) | 0.49(0.07-3.5) | 0.48 |
|  | **dom** | 95 | 50(52.63) | 1.04(0.76-1.42) | 0.80 |  | 61 | 27(44.26) | 1.03(0.68-1.57) | 0.89 |
|  |  |  |  |  |  |  |  |  |  |  |
| **rs2304204** | **A/A** | 249 | 122(49.00) | 1 |  |  | 175 | 74(42.29) | 1 |  |
|  | **A/G** | 188 | 95(50.53) | 0.99(0.75-1.29) | 0.92 |  | 120 | 45(37.50) | 0.81(0.56-1.18) | 0.28 |
|  | **G/G** | 40 | 22(55.00) | 1.18(0.75-1.85) | 0.49 |  | 27 | 11(40.74) | 0.99(0.53-1.87) | 0.99 |
|  | **dom** | 228 | 117(51.32) | 1.02(0.79-1.31) | 0.90 |  | 147 | 56(38.10) | 0.84(0.6-1.2) | 0.34 |
|  |  |  |  |  |  |  |  |  |  |  |
| **rs11770589** | **G/G** | 142 | 79(55.63) | 1 |  |  | 89 | 39(43.82) | 1 |  |
|  | **A/G** | 210 | 106(50.48) | 0.83(0.62-1.11) | 0.21 |  | 81 | 32(39.51) | 0.86(0.54-1.37) | 0.52 |
|  | **A/A** | 109 | 49(44.95) | 0.67(0.47-0.96) | **0.03** |  | 140 | 58(41.43) | 0.93(0.62-1.4) | 0.73 |
|  | **dom** | 319 | 155(48.59) | 0.77(0.59-1.01) | 0.06 |  | 221 | 90(40.72) | 0.9(0.62-1.32) | 0.59 |
|  |  |  |  |  |  |  |  |  |  |  |
| **RS1874327** | **T/T** | 203 | 102(50.25) | 1 |  |  | 132 | 54(40.91) | 1 |  |
|  | **A/T** | 209 | 106(50.72) | 0.97(0.74-1.27) | 0.82 |  | 42 | 19(45.24) | 1.30(0.77-2.2) | 0.32 |
|  | **A/A** | 60 | 32(53.33) | 1.23(0.83-1.84) | 0.30 |  | 145 | 59(40.69) | 0.99(0.68-1.43) | 0.94 |
|  | **dom** | 269 | 138(51.30) | 1.02(0.79-1.32) | 0.88 |  | 187 | 78(41.71) | 1.05(0.74-1.48) | 0.79 |
|  |  |  |  |  |  |  |  |  |  |  |
| **rs2070197** | **T/T** | 372 | 181(48.66) | 1 |  |  | 279 | 114(40.86) | 1 |  |
|  | **C/T** | 101 | 60(59.41) | 1.25(0.93-1.67) | 0.14 |  | 69 | 34(49.28) | 1.17(0.8-1.71) | 0.43 |
|  | **C/C** | 3 | 2(66.67) | 3.09(0.76-12.53) | 0.11 |  | 2 | 2(100.00) | 2.76(0.68-11.2) | 0.16 |
|  | **dom** | 104 | 62(59.62) | 1.27(0.95-1.7) | 0.10 |  | 71 | 36(50.7) | 1.21(0.83-1.75) | 0.33 |
|  |  |  |  |  |  |  |  |  |  |  |
| **rs1061502** | **T/T** | 273 | 140(51.28) | 1 |  |  | 182 | 74(40.66) | 1 |  |
|  | **C/T** | 148 | 75(50.68) | 1.05(0.79-1.38) | 0.76 |  | 26 | 10(38.46) | 0.96(0.49-1.85) | 0.89 |
|  | **C/C** | 32 | 11(34.38) | 0.57(0.31-1.06) | 0.08 |  | 98 | 39(39.80) | 1(0.68-1.47) | 0.98 |
|  | **dom** | 180 | 86(47.78) | 0.95(0.72-1.24) | 0.69 |  | 124 | 49(39.52) | 0.99(0.69-1.42) | 0.94 |

^1^Number of cases may differ due to missing data.

No., number of patients; M=0, no distant metastasis present; HR, hazard ratio, CI, confidence interval

Bold numbers indicate a statistical significance at 5% level.
