# Supplementary material for: Single Nucleotide Polymorphisms within Interferon Signaling Pathway Genes Are Associated with Colorectal Cancer Susceptibility and Survival
Source: PLoS One. 2014 Oct 28;9(10):e111061. doi: 10.1371/journal.pone.0111061 (PMC4211713; doi:10.1371/journal.pone.0111061)
Supplement: Table S4 — Stratified analysis of rs6475526, rs7047687 and rs11770589 for overall survival and rs6475526 for event-free survival among patients without distant metastasis at the time of diagnosis. (DOC) [file pone.0111061.s005.doc]

**Table S4**. Stratified analysis of rs6475526, rs7047687 and rs11770589 for overall survival and rs6475526 for event-free survival among patients without distant metastasis at the time of diagnosis

|  |  | **No.^1^** | **No.^1^ died (%)** | **HR (95%CI)** | ***P* value** | **No.^1^** | **No.^1^ died (%)** | **HR (95%CI)** | ***P* value** |
| --- | --- | --- | --- | --- | --- | --- | --- | --- | --- |
| **Overall survival** | |  |  |  |  |  |  |  |  |
| **Stage1** |  |  |  |  |  | **Stage2** |  |  |  |
| **rs6475526** | **C/C** | 23 | 3(13.04) | 1 |  | 48 | 14(29.17) | 1 |  |
|  | **C/T** | 24 | 9(37.50) | **3.82(1.03-14.12)** | **0.045** | 54 | 18(33.33) | 1.29(0.64-2.61) | 0.476 |
|  | **T/T** | 5 | 2(40.00) | 5.57(0.92-33.63) | 0.062 | 21 | 4(19.05) | 0.71(0.23-2.16) | 0.544 |
|  | **dom** | 29 | 11(37.93) | **4.04(1.13-14.53)** | **0.032** | 75 | 22(29.33) | 1.12(0.57-2.20) | 0.736 |
|  |  |  |  |  |  |  |  |  |  |
| **rs7047687** | **A/A** | 18 | 9(50.00) | 1 |  | 46 | 11(23.91) | 1 |  |
|  | **A/C** | 17 | 4(23.53) | 0.48(0.15-1.55) | 0.218 | 47 | 15(31.91) | 1.20(0.55-2.63) | 0.641 |
|  | **C/C** | 17 | 2(11.76) | **0.17(0.04-0.77)** | **0.022** | 30 | 9(30.00) | 1.14(0.47-2.76) | 0.773 |
|  | **dom** | 34 | 6(17.65) | **0.29(0.10-0.83)** | **0.020** | 77 | 24(31.17) | 1.18(0.58-2.42) | 0.653 |
|  |  |  |  |  |  |  |  |  |  |
| **rs11770589** | **G/G** | 15 | 4(26.67) | 1 |  | 38 | 10(26.32) | 1 |  |
|  | **A/G** | 26 | 10(38.46) | 1.08(0.34-3.44) | 0.900 | 55 | 17(30.91) | 1.27(0.58-2.77) | 0.551 |
|  | **A/A** | 11 | 1(9.09) | 0.17(0.02-1.53) | 0.114 | 29 | 8(27.59) | 0.93(0.37-2.36) | 0.880 |
|  | dom | 37 | 11(29.73) | 0.73(0.23-2.28) | 0.584 | 84 | 25(29.76) | 1.14(0.55-2.37) | 0.733 |
|  |  |  |  |  |  |  |  |  |  |
| **Stage3** |  |  |  |  |  | **Stage4** |  |  |  |
| **rs6475526** | **C/C** | 36 | 13(36.11) | 1 |  | 52 | 41(78.85) | 1 |  |
|  | **C/T** | 65 | 28(43.08) | 1.35(0.70-2.61) | 0.368 | 60 | 57(95.00) | 1.39(0.93-2.09) | 0.107 |
|  | **T/T** | 15 | 7(46.67) | 1.52(0.61-3.83) | 0.372 | 14 | 12(85.71) | 1.22(0.64-2.33) | 0.545 |
|  | **dom** | 80 | 35(43.75) | 1.38(0.73-2.62) | 0.318 | 74 | 69(93.24) | 1.36(0.92-2.01) | 0.121 |
|  |  |  |  |  |  |  |  |  |  |
| **rs7047687** | **A/A** | 47 | 25(53.19) | 1 |  | 48 | 43(89.58) | 1 |  |
|  | **A/C** | 42 | 17(40.48) | 0.69(0.37-1.27) | 0.232 | 42 | 38(90.48) | 1.25(0.81-1.95) | 0.316 |
|  | **C/C** | 30 | 13(43.33) | 0.65(0.33-1.26) | 0.201 | 31 | 24(77.42) | 0.63(0.38-1.05) | 0.076 |
|  | **dom** | 72 | 30(41.67) | 0.67(0.39-1.14) | 0.137 | 73 | 60(82.19) | 0.91(0.62-1.34) | 0.633 |
|  |  |  |  |  |  |  |  |  |  |
| **rs11770589** | **G/G** | 36 | 18(50.00) | 1 |  | 43 | 37(86.05) | 1 |  |
|  | **A/G** | 50 | 18(36.00) | 0.57(0.30-1.11) | 0.098 | 56 | 49(87.5.0) | 0.92(0.60-1.41) | 0.704 |
|  | **A/A** | 32 | 18(56.25) | 1.09(0.57-2.10) | 0.796 | 20 | 17(85.00) | 0.61(0.34-1.08) | 0.088 |
|  | dom | 82 | 36(43.90) | 0.76(0.43-1.34) | 0.334 | 76 | 66(86.84) | 0.81(0.54-1.22) | 0.314 |
|  |  |  |  |  |  |  |  |  |  |
| **Colon** |  |  |  |  |  | **Rectal** |  |  |  |
| **rs6475526** | **C/C** | 112 | 56(50.00) | 1 |  | 64 | 24(37.50) | 1 |  |
|  | **C/T** | 141 | 73(51.77) | 1.11(0.78-1.57) | 0.554 | 83 | 52(62.65) | **2.15(1.32-3.49)** | **0.002** |
|  | **T/T** | 36 | 14(38.89) | 0.70(0.39-1.25) | 0.227 | 29 | 14(48.28) | **1.95(1.01-3.78)** | **0.048** |
|  | **dom** | 177 | 87(49.15) | 1.01(0.72-1.42) | 0.937 | 112 | 66(58.93) | **2.10(1.31-3.36)** | **0.002** |
|  |  |  |  |  |  |  |  |  |  |
| **rs7047687** | **A/A** | 107 | 58(54.21) | 1 |  | 74 | 43(58.11) | 1 |  |
|  | **A/C** | 104 | 49(47.12) | 0.87(0.59-1.27) | 0.474 | 59 | 31(52.54) | 0.79(0.50-1.26) | 0.327 |
|  | **C/C** | 74 | 36(48.65) | 0.78(0.51-1.18) | 0.241 | 46 | 20(43.48) | **0.57(0.33-0.96)** | **0.036** |
|  | **dom** | 178 | 85(47.75) | 0.83(0.59-1.16) | 0.272 | 105 | 51(48.57) | 0.69(0.46-1.03) | 0.069 |
|  |  |  |  |  |  |  |  |  |  |
| **rs11770589** | **G/G** | 91 | 51(56.04) | 1 |  | 51 | 28(54.90) | 1 |  |
|  | **A/G** | 125 | 64(51.20) | 0.81(0.56-1.17) | 0.266 | 85 | 42(49.41) | 0.87(0.54-1.40) | 0.564 |
|  | **A/A** | 72 | 29(40.28) | **0.56(0.36-0.89)** | **0.014** | 37 | 20(54.05) | 0.91(0.52-1.62) | 0.759 |
|  | dom | 197 | 93(47.21) | 0.71(0.51-1.00) | 0.053 | 122 | 62(50.82) | 0.88(0.57-1.38) | 0.585 |
|  |  |  |  |  |  |  |  |  |  |
| **Grade 1,2** |  |  |  |  |  | **Grade 3,4** |  |  |  |
| **rs6475526** | **C/C** | 116 | 49(42.24) | 1 |  | 36 | 18(50.00) | 1 |  |
|  | **C/T** | 145 | 71(48.97) | 1.26(0.87-1.81) | 0.218 | 44 | 26(59.09) | 1.50(0.82-2.75) | 0.187 |
|  | **T/T** | 36 | 15(41.67) | 1.12(0.63-2.00) | 0.701 | 22 | 10(45.45) | 0.89(0.41-1.94) | 0.775 |
|  | **dom** | 181 | 86(47.51) | 1.23(0.87-1.75) | 0.246 | 66 | 36(54.55) | 1.26(0.72-2.22) | 0.424 |
|  |  |  |  |  |  |  |  |  |  |
| **rs7047687** | **A/A** | 109 | 58(53.21) | 1 |  | 44 | 23(52.27) | 1 |  |
|  | **A/C** | 103 | 45(43.69) | 0.78(0.53-1.16) | 0.221 | 34 | 17(50.00) | 0.88(0.47-1.65) | 0.684 |
|  | **C/C** | 83 | 35(42.17) | 0.67(0.44-1.02) | 0.064 | 25 | 14(56.00) | 0.90(0.46-1.74) | 0.745 |
|  | **dom** | 186 | 80(43.01) | 0.73(0.52-1.03) | 0.070 | 59 | 31(52.54) | 0.89(0.52-1.52) | 0.660 |
|  |  |  |  |  |  |  |  |  |  |
| **rs11770589** | **G/G** | 87 | 43(49.43) | 1 |  | 37 | 21(56.76) | 1 |  |
|  | **A/G** | 142 | 66(46.48) | 0.83(0.56-1.22) | 0.335 | 43 | 22(51.16) | 0.94(0.52-1.71) | 0.842 |
|  | **A/A** | 69 | 30(43.48) | 0.74(0.46-1.18) | 0.201 | 20 | 9(45.00) | 0.69(0.31-1.51) | 0.353 |
|  | dom | 211 | 96(45.50) | 0.80(0.56-1.14) | 0.217 | 63 | 31(49.21) | 0.85(0.49-1.49) | 0.572 |
|  |  |  |  |  |  |  |  |  |  |
| **M0** |  |  |  |  |  | **M1** |  |  |  |
| **rs6475526** | **C/C** | 115 | 34(29.57) | 1 |  | 52 | 41(78.85) | 1 |  |
|  | **C/T** | 149 | 58(38.93) | **1.55(1.01-2.37)** | **0.044** | 60 | 57(95.00) | 1.39(0.93-2.09) | 0.107 |
|  | **T/T** | 46 | 15(32.61) | 1.29(0.70-2.37) | 0.411 | 14 | 12(85.71) | 1.22(0.64-2.33) | 0.545 |
|  | **dom** | 195 | 73(37.44) | 1.49(0.99-2.24) | 0.057 | 74 | 69(93.24) | 1.36(0.92-2.01) | 0.121 |
|  |  |  |  |  |  |  |  |  |  |
| **rs7047687** | **A/A** | 118 | 49(41.53) | 1 |  | 48 | 43(89.58) | 1 |  |
|  | **A/C** | 112 | 37(33.04) | 0.74(0.48-1.13) | 0.161 | 42 | 38(90.48) | 1.25(0.81-1.95) | 0.316 |
|  | **C/C** | 83 | 28(33.73) | 0.68(0.43-1.09) | 0.108 | 31 | 24(77.42) | 0.63(0.38-1.05) | 0.076 |
|  | **dom** | 195 | 65(33.33) | 0.71(0.49-1.03) | 0.074 | 73 | 62(84.93) | 0.91(0.62-1.34) | 0.633 |
|  |  |  |  |  |  |  |  |  |  |
| **rs11770589** | **G/G** | 89 | 34(38.2) |  |  | 43 | 37(86.05) | 1 |  |
|  | **A/G** | 140 | 49(35.00) | 0.86(0.56-1.34) | 0.510 | 56 | 49(87.5) | 0.92(0.60-1.41) | 0.704 |
|  | **A/A** | 81 | 29(35.80) | 0.87(0.53-1.42) | 0.575 | 20 | 17(85.00) | 0.61(0.34-1.08) | 0.088 |
|  | dom | 221 | 78(35.29) | 0.87(0.58-1.29) | 0.480 | 76 | 66(86.84) | 0.81(0.54-1.22) | 0.314 |
|  |  |  |  |  |  |  |  |  |  |
| **Event-free survival (M=0)** | | |  |  |  |  |  |  |  |
|  |  | **No.^1^** | **No.^1^ event (%)** | **HR (95%CI)** | ***P* value** | **No.^1^** | **No.^1^ event (%)** | **HR (95%CI)** | ***P* value** |
| **Stage1** |  |  |  |  |  | **Stage2** |  |  |  |
| **rs6475526** | **C/C** | 23 | 4(17.39) | 1 |  | 48 | 16(33.33) | 1 |  |
|  | **C/T** | 24 | 10(41.67) | **3.35(1.05-10.72)** | **0.042** | 54 | 22(40.74) | 1.39(0.73-2.65) | 0.320 |
|  | **T/T** | 5 | 3(60.00) | **7.05(1.53-32.60)** | **0.012** | 21 | 5(23.81) | 0.79(0.29-2.16) | 0.649 |
|  | **dom** | 29 | 13(44.83) | **3.78(1.23-11.66)** | **0.021** | 75 | 27(36.00) | 1.22(0.66-2.26) | 0.534 |
|  |  |  |  |  |  |  |  |  |  |
| **Stage3** |  |  |  |  |  |  |  |  |  |
| **rs6475526** | **C/C** | 36 | 14(38.89) | 1 |  |  |  |  |  |
|  | **C/T** | 65 | 32(49.23) | 1.49(0.79-2.79) | 0.217 |  |  |  |  |
|  | **T/T** | 15 | 7(46.67) | 1.48(0.60-3.66) | 0.400 |  |  |  |  |
|  | **dom** | 80 | 39(48.75) | 1.48(0.81-2.73) | 0.205 |  |  |  |  |
|  |  |  |  |  |  |  |  |  |  |
| **Colon** |  |  |  |  |  | **Rectal** |  |  |  |
| **rs6475526** | **C/C** | 68 | 23(33.82) | 1 |  | 47 | 16(34.04) | 1 |  |
|  | **C/T** | 90 | 37(41.11) | 1.36(0.81-2.29) | 0.245 | 59 | 31(52.54) | **1.96(1.07-3.59)** | **0.030** |
|  | **T/T** | 25 | 8(32.00) | 0.96(0.43-2.16) | 0.928 | 21 | 9(42.86) | 1.72(0.76-3.91) | 0.193 |
|  | **dom** | 115 | 45(39.13) | 1.27(0.77-2.10) | 0.354 | 80 | 40(50.00) | **1.90(1.06-3.40)** | **0.031** |
|  |  |  |  |  |  |  |  |  |  |
| **Grade 1,2** |  |  |  |  |  | **Grade 3,4** |  |  |  |
| **rs6475526** | **C/C** | 84 | 29(34.52) | 1 |  | 20 | 12(60.00) | 1 |  |
|  | **C/T** | 112 | 49(43.75) | 1.41(0.89-2.24) | 0.141 | 21 | 11(52.38) | 1.58(0.62-4.02) | 0.339 |
|  | **T/T** | 26 | 7(26.92) | 0.80(0.35-1.82) | 0.587 | 17 | 9(52.94) | 1.58(0.59-4.24) | 0.361 |
|  | **dom** | 138 | 56(40.58) | 1.29(0.82-2.02) | 0.270 | 38 | 20(52.63) | 1.58(0.68-3.65) | 0.284 |

^1^Number of cases may differ due to missing data.

No., number of patients; M, presence of metastasis; M=0, no distant metastasis present; HR, hazard ratio; CI, confidence interval.

Bold numbers indicate a statistical significance at 5% level.
